# Motor-Derived Digital Biomarkers for Identifying Low-MoCA Status in People with Parkinson’s Disease

**DOI:** 10.3390/s26082503

**Published:** 2026-04-18

**Authors:** Bohyun Kim, Changhong Youm, Sang-Myung Cheon, Hwayoung Park, Hyejin Choi, Juseon Hwang, Minsoo Kim

**Affiliations:** 1 Biomechanics Laboratory, Dong-A University, Saha-gu, Busan 49315, Republic of Korea; 2177638@donga.ac.kr (B.K.); app00113@donga.ac.kr (H.P.); chjin0907@donga.ac.kr (H.C.); 2577561@donga.ac.kr (J.H.); 2677610@donga.ac.kr (M.K.); 2 Department of Health Sciences, The Graduate School, Dong-A University, Saha-gu, Busan 49315, Republic of Korea; 3 Department of Neurology, School of Medicine, Dong-A University, Seo-gu, Busan 49201, Republic of Korea; smcheon@dau.ac.kr

**Keywords:** Parkinson’s disease, cognitive decline, digital biomarkers, multi-feature analysis, machine learning, wearable sensors

## Abstract

**Highlights:**

**What are the main findings?**

- Specific motor parameters—global motor severity, stride length during turning, and ankle jerk amplitude—significantly predict cognitive performance in Parkinson’s disease.
- A compact set of motor-derived digital biomarkers classified low-MoCA status with 65.8% accuracy (AUC = 0.737) without direct cognitive testing.

**What are the implications of the main findings?**

- Quantitative gait and motor metrics may enable early screening of cognitive vulnerability in Parkinson’s disease using routine motor assessments.
- Motor-derived digital biomarkers could support objective, scalable tools for cognitive risk identification in clinical and remote monitoring settings.

**Abstract:**

Cognitive impairment is a prevalent non-motor manifestation of Parkinson’s disease (PD), yet early detection remains limited by the sensitivity of conventional cognitive assessments. Emerging evidence suggests that motor dysfunction, particularly gait and balance abnormalities, reflects underlying cognitive vulnerability. This study examined motor–cognitive associations and evaluated whether motor-derived features can be used to classify low-MoCA status in PD without direct cognitive testing. Data from 102 individuals with PD were analyzed, incorporating clinical assessments, physical function measures, lifestyle factors, and gait-derived biomarkers. Multiple regression identified Unified Parkinson’s Disease Rating Scale Part III, stride length of the more affected side during 360° turning at preferred speed, and maximum ankle jerk on the less affected side during forward walking as independent predictors of Montreal Cognitive Assessment scores, collectively explaining 34.7% of the variance. Network analysis revealed integrative relationships among global motor severity, gait smoothness, and cognitive performance. Using a compact motor-based feature set, logistic regression achieved a mean accuracy of 65.8% and an AUC of 0.737 in classifying low-MoCA status under cross-validation. These findings demonstrate that motor-derived digital biomarkers capture clinically meaningful information about cognitive status in PD and may serve as adjunctive tools for identifying cognitive vulnerability in clinical settings.

## 1. Background

Parkinson’s disease (PD) is a progressive neurodegenerative disorder characterized by both motor and non-motor symptoms, which substantially impair quality of life in affected individuals [1]. While hallmark motor symptoms include bradykinesia, rigidity, resting tremor, and postural instability, non-motor symptoms—particularly cognitive impairment—can be equally disabling and may even precede the onset of motor symptoms [2]. Approximately 42.5% of newly diagnosed individuals with PD present with mild cognitive impairment, with 70–80% progressing to dementia over 15–20 years [3,4]. Recent studies have increasingly focused on predicting cognitive decline in PD by integrating clinical evaluations, biomarkers, neuropsychological tests, and longitudinal data [5,6,7,8,9,10,11,12]. These methods promote early identification, timely therapeutic intervention, and enhanced disease management [13].

Various cognitive assessment scales have been utilized to identify individuals with lower cognitive status in PD, and several studies have investigated the predictive abilities of these approaches [14,15]. The Montreal Cognitive Assessment (MoCA), though widely adopted for evaluating memory, executive function, and verbal fluency in PD [5,8,9,16,17], is limited by its intermittent administration, potentially failing to detect subtle or early cognitive changes [18]. Emerging evidence suggests a link between motor and cognitive symptoms in PD, mediated by overlapping neuropathology in the basal ganglia and prefrontal dopaminergic circuits [19]. Specifically, gait disturbances, postural instability, and impaired turning performance have been associated with cognitive decline, indicating that movement-based digital metrics could serve as early biomarkers [20,21].

Recent advances in digital healthcare have enabled the use of wearable sensors for noninvasive evaluation of gait impairments in individuals with PD [22,23,24]. Metrics derived from these sensors—such as stride variability, turning angular velocity, and entropy-based indicators of gait regularity—have proven sensitive in detecting subtle cognitive-motor dysfunctions often overlooked by traditional clinical scales [23,25,26]. These devices offer continuous, real-world gait monitoring, providing high-resolution, objective data that address key limitations of conventional assessments [27]. Previous studies relying exclusively on standard clinical tools have demonstrated poor reproducibility across patient cohorts, highlighting the need to incorporate objective, multi-feature data to enhance the prediction and classification of cognitive decline in PD [28,29].

Machine learning (ML) techniques have emerged as effective tools for integrating high-dimensional, heterogeneous multi-feature data, enabling the detection of complex motor–cognitive interactions and the systematic selection of predictive features [28,30,31]. Recent studies have demonstrated that techniques such as regularized regression and tree-based models can aid in identifying candidate digital biomarkers related to disease severity and progression [28,30,32]. However, most existing ML models either insufficiently incorporate sensor-derived gait features or inadequately examine their direct relationship with cognitive decline in PD. Moreover, despite their promise as screening tools, current digital biomarker models often lack interpretability and clinical utility [33]. Accordingly, there is a growing need for analytical frameworks that prioritize interpretability and physiological relevance, integrating gait-derived digital metrics with conventional clinical and physical function measures. Such approaches may provide more clinically meaningful insights into motor–cognitive interactions and support the identification of cognitive vulnerability in PD beyond reliance on standalone cognitive screening tools.

Despite recent progress, critical gaps remain in incorporating gait features derived from wearable sensors into clinically interpretable models of cognitive impairment in PD. This study aims to enhance the accuracy of predicting and classifying low-MoCA status in PD by applying ML techniques to multi-feature data, including clinical characteristics, physical function, lifestyle factors, and gait-derived features. Specifically, the objectives are as follows:

1. Investigate the associations between multi-features—such as clinical characteristics, physical function, lifestyle factors, and gait-derived features—and low-MoCA status in individuals with PD.
2. Perform stepwise multiple linear regression to identify independent predictors of cognitive performance.
3. Explore relational patterns among multi-features using network-based visualization.
4. Assess logistic regression models to classify individuals with and without cognitive impairment based on selected multi-features.

We hypothesize that multi-features—particularly gait-derived digital biomarkers—will show significant associations with cognitive status in PD. Furthermore, integrating these features with ML techniques is expected to improve MoCA score prediction and the classification of cognitive impairment, offering insights that extend beyond conventional clinical assessments.

## 2. Methods

### 2.1. Participants

Data from 102 individuals with a clinician-confirmed diagnosis of idiopathic PD were included, based on the UK Parkinson’s Disease Society Brain Bank Clinical Diagnostic Criteria. Inclusion criteria required participants to have mild-to-moderate idiopathic PD, be on anti-Parkinsonian medication, and be able to stand and walk independently during clinical assessments. Exclusion criteria included any comorbid neurological, orthopedic, or psychiatric disorders. The participants had a mean age of 68.1 years, and 54.9% were female. The distribution of Hoehn and Yahr stages among participants was as follows: 27.5% in stage 1, 49.0% in stage 2, and 23.5% in stage 3, reflecting a range from mild to moderate disease severity. The mean disease duration was 5.7 years, and the average motor examination subscale score of the Unified Parkinson’s Disease Rating Scale (UPDRS) was 29.4. Participants were classified into two groups based on MoCA scores (<26): a normal-MoCA group (n = 60) and a low-MoCA group (*n* = 42) (Table 1, Supplementary Table S1).

The study was approved by the Institutional Review Board of Dong-A University Medical Center (Approval No. DAUHIRB-22-089). All participants were fully informed of the study purpose and procedures and provided written informed consent. The study was registered with the Clinical Research Information Service in the Republic of Korea (KCT0009353).

### 2.2. Experimental Procedures

**Figure 1:**
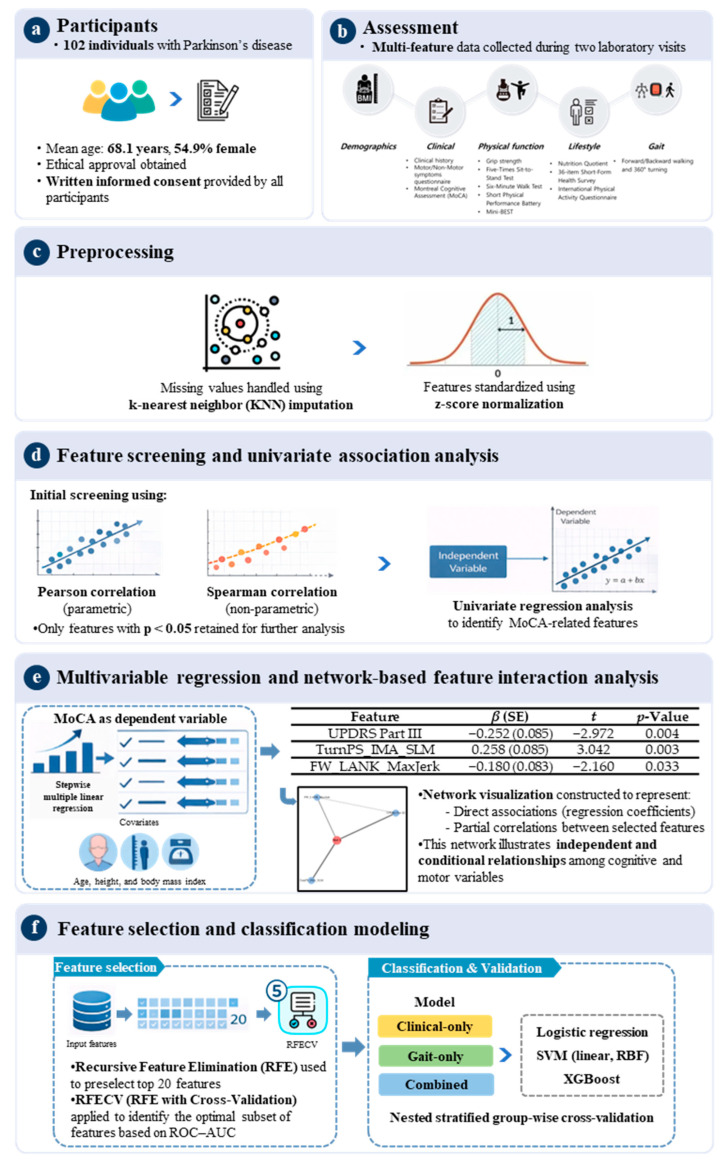
Workflow for classifying low-MoCA status in PD and identifying digital biomarkers.

Participants completed two laboratory visits for comprehensive multi-feature evaluation, including assessments of demographic and clinical characteristics, physical function, lifestyle factors, and gait parameters (Figure 1). All participants were receiving dopaminergic therapy and were assessed during the “ON” medication state, approximately 2–3 h after dosing. The levodopa-equivalent daily dose (LEDD) was recorded for all subjects. However, owing to the uniform medication status and the absence of a significant correlation between LEDD and MoCA scores (Spearman ρ = −0.066, *p* = 0.510), LEDD was not included as a covariate in the predictive models.

The study assessed the following categories of multi-features. Clinical measurements included 21 features, with PD severity evaluated using the Hoehn and Yahr scale and the UPDRS comprising total and Parts I–IV scores. Part I assessed non-motor aspects of daily living, including cognitive impairment and mood disturbances. Part II measured motor-related daily activities, such as speech, handwriting, and hygiene. Part III evaluated motor symptom severity, focusing on tremors, rigidity, and bradykinesia. Part IV captured motor complications related to long-term dopaminergic therapy, such as dyskinesia and motor fluctuations. Clinical history, including symptom and treatment duration and LEDD, was collected to reflect disease progression and pharmacological management. Cognitive function was assessed using MoCA. MoCA scores were adjusted for education level by adding 1 point for participants with ≤6 years of education, in accordance with standard guidelines. The Beck Depression Inventory and Beck Anxiety Inventory were used to measure depressive and anxiety symptoms, respectively. The PD Sleep Scale-2 assessed sleep disturbances, and the Epworth Sleepiness Scale evaluated daytime sleepiness. Fatigue severity was measured using the Fatigue Severity Scale. Fall risk and fear of falling were evaluated based on fall history and the Korean version of the Falls Efficacy Scale. Self-efficacy for exercise was assessed using the Self-Efficacy for Exercise Scale. The New Freezing of Gait Questionnaire measured the severity of freezing episodes. The Non-Motor Symptoms Scale provided a comprehensive evaluation of a range of non-motor symptoms. Lastly, the PD Questionnaire-39 assessed health-related quality of life.

The physical function and lifestyle assessments comprised 10 features. Grip strength was measured bilaterally, along with performance-based tests, including the Five-Times Sit-to-Stand Test, the Six-Minute Walk Test, the Short Physical Performance Battery, and the Mini-Balance Evaluation Systems Test (Mini-BEST). Lifestyle-related measures included the Nutrition Quotient; the 36-item Short-Form Health Survey, which provided total, physical, and mental health scores; and the International Physical Activity Questionnaire.

Gait-derived features obtained from motion capture and wearable sensors comprised 38 and 360 features, respectively. Participants completed three walking tasks: (1) forward and (2) backward straight walking at a self-selected speed, and (3) 360° turns at self-selected and maximum speeds in the direction of the inner step of the more affected side and the outer step of the more affected side. Each measurement was repeated three times, and the mean value was calculated.

Spatiotemporal gait features were extracted from three-dimensional marker trajectories (Plug-in Gait model, Vicon Motion Systems, UK; sampling rate = 100 Hz). Marker trajectories were filtered using a zero-lag fourth-order low-pass Butterworth filter with a cutoff frequency of 6 Hz to reduce high-frequency noise. Extracted spatiotemporal parameters included walking speed (m/s), stride length (m), single- and double-support phases (%), and contralateral temporal coordination. The more affected–less affected side contralateral temporal coordination was defined as the average difference between the more affected side of the elbow and the less affected side of the knee. The less affected–more affected side contralateral temporal coordination was defined as the average difference between the less affected side of the elbow and the more affected side of the knee.

Wearable sensor data were collected using six inertial measurement units (Xsens DOT, Movella Technologies, Netherlands) placed at anatomically defined locations: more and less affected sides of the upper arms (MELB, LELB; 5 cm above the lateral humeral epicondyle), more and less affected side of lateral malleolus (MANK, LANK), 10th thoracic spine (T10), and lumbar spine (center of the left and right posterior superior iliac spine, PSIS). Each IMU recorded triaxial acceleration and angular velocity at 60 Hz, aligned to anatomical axes (x: vertical, y: mediolateral, z: anteroposterior). Wearable sensor-derived features encompassed maximum jerk and angular velocity jerk, mean and maximum acceleration, mean and maximum gyroscope values, root-mean-square (RMS) acceleration and gyroscope values, and sample entropy of acceleration and gyroscope signals (Table 2). Prior to feature extraction, raw IMU signals were filtered using a low-pass Butterworth filter to reduce high-frequency noise, and segmented into task-specific gait cycles based on gait event detection. Time-derivative features were computed using numerical differentiation based on finite differences. Jerk was defined as the first derivative of linear acceleration with respect to time, and angular jerk was defined as the second derivative of angular velocity. All magnitude-based features were computed using the Euclidean norm of the tri-axial signals. Sample entropy was calculated to quantify movement complexity using parameters m = 2 and r = 0.2 × standard deviation of the signal, following established conventions.

### 2.3. Preprocessing

The dataset was first reviewed to assess its structure and identify inconsistencies. The proportion and pattern of missing data were examined prior to preprocessing. Overall, missingness was low and did not exceed <5% for any individual feature, and missing values were assumed to be missing at random. Missing values were addressed using k-nearest neighbors’ imputation (k = 5), with each missing value estimated based on the mean of the five most similar observations. Following imputation, z-score normalization was applied to standardize features for exploratory and regression analyses. No additional imputation was required for the classification analyses, as the dataset did not contain missing values after preprocessing.

### 2.4. Feature Screening and Univariate Association Analysis

All candidate variables included demographic characteristics, clinical measures, physical function assessments, lifestyle factors, and gait-derived features. To ensure that the analysis reflected motor and non-cognitive correlates of cognitive status, direct cognitive test scores (i.e., MoCA total score and subdomains) were excluded from all feature screening, regression, and classification procedures.

As an initial screening step, correlation analyses were performed to identify motor-related features potentially associated with cognitive performance, as measured by MoCA scores. Pearson correlation coefficients were computed for variables with approximately normal distributions, while Spearman rank correlations were used for non-normally distributed variables. This dual approach allowed assessment of both linear and monotonic associations. Features demonstrating statistically significant correlations with MoCA scores (*p* < 0.05) were retained for subsequent analyses.

To further evaluate the independent associations between features that demonstrated statistically significant correlations in the initial screening step and cognitive performance, univariate linear regression analyses were conducted. Each feature was entered separately into a regression model with MoCA score as the dependent variable, adjusting for age, height, and body mass index (BMI). Variables showing statistically significant associations (*p* < 0.05) were considered candidates for multivariable modeling.

### 2.5. Multivariable Regression Analysis

To examine the combined and independent contributions of motor-related features to cognitive performance, stepwise multiple linear regression analysis was performed using the features retained from the univariate analyses. Age, height, and BMI were included as covariates to account for potential confounding effects. Stepwise selection was applied to identify a parsimonious set of predictors that significantly explained variance in MoCA scores. Multicollinearity was assessed using variance inflation factors (VIFs), with values below 1.3 considered indicative of acceptable collinearity.

### 2.6. Network-Based Feature Interaction Analysis

Network-based visualization was employed to explore the relational structure among cognitive performance and selected features identified through multivariable regression analysis [34,35]. Features retained in the final regression model were represented as nodes, while edges represented associations between nodes derived from regression coefficients and partial correlations. The dependent variable (MoCA score) and selected features were first incorporated into a network graph. Edges connecting MoCA to each selected feature were weighted according to the absolute value of standardized regression coefficients, reflecting the strength of their direct associations with cognitive performance. To further characterize interrelationships among selected features beyond their direct associations with MoCA, a partial correlation network was estimated using the graphical least absolute shrinkage and selection operator (LASSO) with cross-validated regularization [36]. This approach estimates sparse inverse covariance matrices, enabling identification of conditional dependencies between selected features while accounting for the influence of other features [34]. Partial correlation coefficients were derived from the precision matrix, and edges were retained only when the absolute partial correlation exceeded a predefined threshold (|r| ≥ 0.10), ensuring a parsimonious network structure.

The network was visualized using a force-directed layout algorithm (Fruchterman–Reingold spring layout), whereby nodes with stronger connections were positioned closer together [37]. Node size was scaled according to the magnitude of regression coefficients, and edge thickness reflected the strength of partial correlations. The network-based representation was intended as an exploratory and complementary visualization to illustrate integrative and relational patterns among motor and cognitive features beyond the regression results, rather than to provide independent inferential conclusions or causal interpretations. All network analyses and visualizations were performed using Python-based open-source libraries.

### 2.7. Feature Selection and Classification Modeling

To identify digital motor biomarkers for classifying current cognitive status (low-MoCA), a nested cross-validation framework was implemented. In each outer fold, feature selection was performed exclusively within the training data. First, recursive feature elimination (RFE) with logistic regression was applied to reduce the feature set to a maximum of 20 variables. Subsequently, RFE with cross-validation (RFECV) was conducted within the training fold using logistic regression as the base estimator, optimizing the feature subset based on cross-validated the area under the receiver operating characteristic curve (ROC–AUC). The selected features from each training fold were then applied to the corresponding held-out test fold. Feature standardization was also performed within each training fold using z-score normalization, and the fitted scaling parameters were applied to the test data.

Three classification models were evaluated: (1) logistic regression, (2) support vector machine (SVM) with a linear kernel, (3) SVM with a radial basis function (RBF) kernel, and XGBoost. The selection of classification algorithms was guided by the study objective of identifying clinically interpretable digital biomarkers rather than maximizing predictive performance alone. Logistic regression was selected as the primary model due to its interpretability and ability to provide direct insights into feature contributions. SVM were included to evaluate both linear and nonlinear decision boundaries and to assess potential performance improvements beyond linear models. XGBoost was additionally incorporated as a nonlinear ensemble benchmark to explore whether more complex models could provide meaningful performance gains in the context of a relatively small sample size and high-dimensional feature space.

Because repeated task trials were averaged at the participant level, each participant contributed a single observation to the classification analysis. To ensure strict separation of participant-level data across training and test folds, stratified group-wise cross-validation (10 folds) was employed using participant identifiers as grouping variables. This approach preserved class balance while preventing any potential overlap of participant-level data between folds. Both outer and inner cross-validation procedures were implemented using stratified group-wise splitting based on participant identifiers. To evaluate the incremental value of sensor-derived features beyond conventional clinical assessments, three model configurations were defined within the same nested cross-validation framework: (1) a clinical-only model, (2) a gait-only model, and (3) a combined model integrating all features. This design enabled direct comparison of the independent and complementary contributions of clinical and gait-derived features.

All model configurations underwent identical preprocessing, feature selection procedures, and validation strategies to ensure fair comparison. Feature selection (RFE and RFECV) was applied independently within each training fold for each model configuration. Model performance was evaluated using accuracy, ROC–AUC, sensitivity, specificity, positive predictive value (PPV), and negative predictive value (NPV). Confidence intervals for clinical metrics were estimated using bootstrap resampling. ROC curves were generated using aggregated predictions across folds. To assess the robustness of feature selection, stability analysis was conducted by calculating the frequency with which each feature was selected across cross-validation folds. Features consistently selected across folds were considered more stable and reliable predictors.

All analyses were implemented in Python using scikit-learn and XGBoost libraries. A fixed random seed (random_state = 42) was used for all model training, cross-validation splitting, and resampling procedures to ensure reproducibility. Feature selection was performed using a two-stage procedure within each training fold. First, RFE was applied with logistic regression (L2 penalty, C = 0.5, solver = ‘liblinear’, max_iter = 10,000) to reduce the feature set to a maximum of 20 variables with a step size of 2. Subsequently, RFECV was conducted using the same logistic regression estimator with a minimum of 5 features, a step size of 2, and 5-fold stratified group-wise cross-validation, optimizing the feature subset based on ROC–AUC. For classification models, logistic regression was implemented with L2 regularization (C = 0.5, solver = ‘liblinear’, max_iter = 10,000). Support vector machines (SVM) were evaluated using both linear and radial basis function (RBF) kernels with C = 1.0 and default gamma settings (‘scale’ for RBF). XGBoost was configured with 100 estimators, a maximum tree depth of 3, a learning rate of 0.05, subsample ratio of 0.8, column subsample ratio of 0.8, L2 regularization (lambda = 1.0), and a binary logistic objective function.

### 2.8. Statistical Analysis

Data normality was assessed using the Shapiro–Wilk test. Depending on the distribution, either an independent *t*-test or a non-parametric test was used to compare physical and clinical characteristics between groups. Statistical analyses were performed using SPSS version 21.0 (IBM Corp., Armonk, NY, USA), with significance set at *p* < 0.05. Additionally, data preprocessing and analysis were conducted in Python (version 3.10.18) using standard libraries, including pandas, NumPy, scikit-learn, and matplotlib.

## 3. Results

### 3.1. Demographic and Clinical Characteristics Associated with Low-MoCA Status in PD

Table 1 presents the demographic and clinical characteristics of the study participants. Individuals were classified into two groups based on MoCA scores (<26): normal-MoCA group (n = 60) and low-MoCA group (n = 42). The analysis revealed significant demographic and clinical differences between the groups. Specifically, individuals in the low-MoCA group were older and had more severe motor symptoms, as indicated by higher Hoehn and Yahr stages and elevated scores on both the total Unified Parkinson’s Disease Rating Scale (UPDRS) and the motor examination subscale (UPDRS Part III). Additionally, the low-MoCA group demonstrated significantly lower scores on cognitive assessments compared with the normal-MoCA group.

### 3.2. Correlation and Univariate Regression Analysis of Multi-Features

To address the high feature-to-sample ratio, a structured multi-stage feature reduction strategy was applied. The initial feature pool consisted of 434 variables. Correlation analysis was performed as an initial screening step to identify motor features potentially associated with MoCA scores. Features showing statistically significant correlations (*p* < 0.05) were subsequently evaluated using univariate linear regression analyses. Univariate linear regression analyses were conducted to further assess the associations between individual motor features and MoCA scores while adjusting for age, height, and BMI. Correlation-based screening reduced this number to 127 features, and univariate regression analyses further identified 19 candidate features. 19 motor features demonstrated statistically significant associations (*p* < 0.05) and were subsequently entered into the multivariable stepwise linear regression model for further refinement (Supplementary Table S2).

### 3.3. Multivariable Regression and Network-Based Analysis of Features Associated with MoCA Scores

Stepwise multiple linear regression analysis was performed to examine the independent contributions of selected features to MoCA scores, with age, height, and BMI included as covariates. The final model explained 34.7% of the variance in MoCA scores (adjusted R^2^ = 0.347) and retained motor examination scores (UPDRS Part III), the stride length of the more affected side (SLM) during 360° turns at the preferred speed in the direction of the inner step of the more affected side (TurnPS_IMA), and the maximum jerk (MaxJerk) of the ankle on the less affected side (LANK) during forward walking (FW). All predictors retained in the final model were statistically significant (*p* < 0.05), with no evidence of multicollinearity (all VIFs < 1.3) (Table 3).

**Figure 2:**
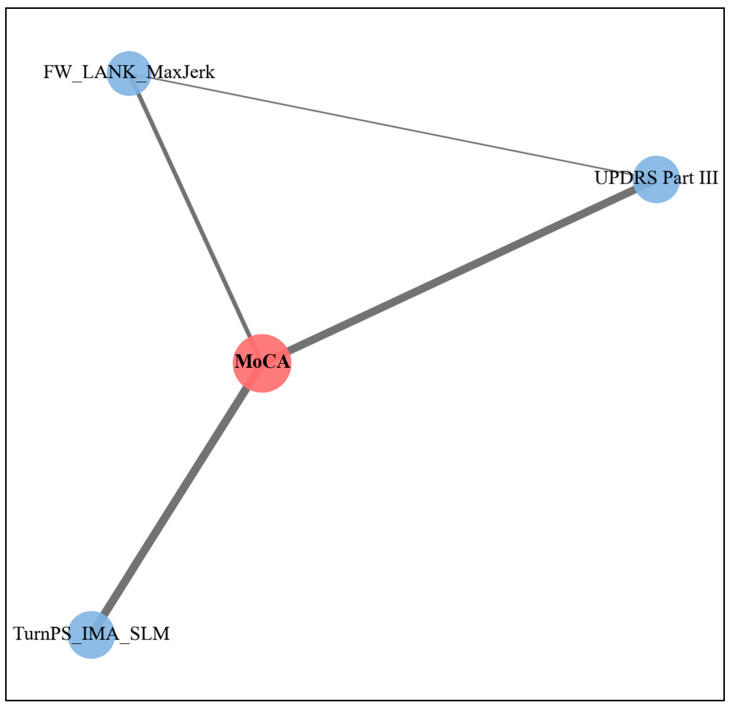
Feature types and predictive weights in the MoCA regression network. Node colors indicate variable types (red: dependent variable; blue: cognitive and gait predictors), and node size reflects the magnitude of standardized regression coefficients. Edge thickness represents the strength of partial correlations, estimated using the graphical LASSO. MoCA, Montreal Cognitive Assessment; UPDRS, Unified Parkinson’s Disease Rating Scale; TurnPS_IMA, 360° turns at preferred speeds in the direction of the inner step of the more affected side; SLM, Stride length of the more affected side; FW, Forward walking; LANK, Less affected side of ankle; MaxJerk, Maximum jerk.

Beyond regression analysis, network-based visualization revealed the relational structure among cognitive, clinical, and motor features. Within the network, UPDRS Part III showed the strongest direct connection to MoCA, consistent with its significant association observed in the regression analysis. Among gait-derived features, TurnPS_IMA_SLM was directly connected to MoCA, supporting its independent association with cognitive performance. In addition, FW_LANK_MaxJerk emerged as a functionally integrative node, linking UPDRS Part III and MoCA with moderate connection strengths, including an edge weight of 0.127 between UPDRS Part III and FW_LANK_MaxJerk (Figure 2).

### 3.4. Identification of Digital Biomarkers Based on Classification Models

In the classification pipeline, RFE retained all 19 variables (limited by the number of candidates), and five stable digital biomarkers for classifying low-MoCA status were identified using RFECV within the nested cross-validation framework. The final biomarker panel included UPDRS Part III, the Mini-BEST, and three gait-derived features (FW_LANK_MaxJerk, SLM during 360° turns performed at the maximum speed in the direction of the inner step of the more affected side (TurnFS_IMA), and the stride length of the less affected side (SLL) during TurnPS_IMA). These features were consistently selected across cross-validation folds, with high selection frequencies observed for key clinical and gait-derived variables, supporting their stability and robustness despite the high feature-to-sample ratio.

To evaluate the incremental contribution of sensor-derived features, we compared clinical-only, gait-only, and combined models within the same nested cross-validation framework (Supplementary Table S3). The clinical-only model demonstrated strong discriminative performance (ROC–AUC ≈ 0.82), indicating that established clinical measures capture substantial information related to cognitive status. The gait-only model showed moderate performance (ROC–AUC ≈ 0.71–0.73), suggesting that wearable-derived features independently reflect aspects of motor–cognitive interaction. The combined model demonstrated comparable performance to the clinical-only model, indicating that sensor-derived features may not substantially increase overall classification accuracy but provide complementary information reflecting distinct motor characteristics associated with low-MoCA status.

The classification performance of the models is summarized in Table 4. Logistic regression demonstrated stable and balanced performance, achieving a mean accuracy of 0.658 ± 0.138 and a mean ROC–AUC of 0.737 ± 0.194. It also achieved a sensitivity of 0.524 (95% CI, 0.370–0.667), specificity of 0.750 (95% CI, 0.636–0.855), PPV of 0.595 (95% CI, 0.424–0.744), and NPV of 0.692 (95% CI, 0.571–0.797). The SVM models demonstrated comparable or slightly improved discriminative performance. The linear SVM achieved a ROC–AUC of 0.761 ± 0.188, while the RBF SVM achieved the highest performance among the evaluated models, with a ROC–AUC of 0.780 ± 0.139 and higher specificity. XGBoost, evaluated as a nonlinear benchmark within the same nested cross-validation framework, demonstrated comparable performance (ROC–AUC = 0.740 ± 0.162) but did not provide a meaningful advantage over the other models. Overall, although nonlinear models showed slightly higher classification performance, logistic regression was selected as the primary model due to its interpretability and clinical relevance.

**Figure 3:**
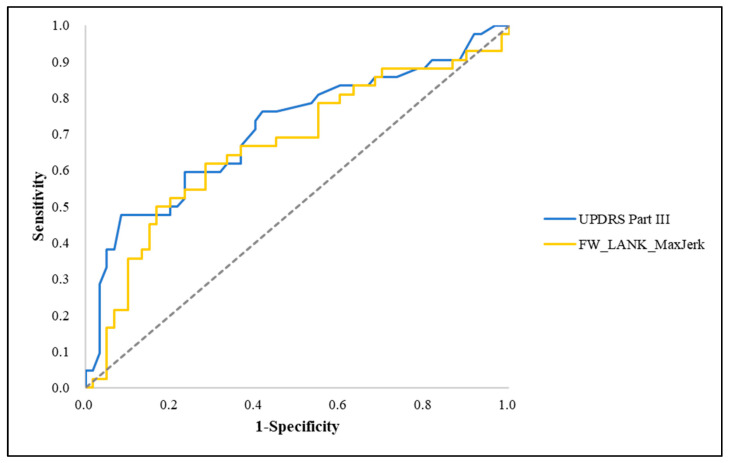
Receiver operating characteristic (ROC) curves for representative individual features associated with MoCA score. These curves illustrate the discriminative ability of single features. The dashed diagonal line represents the line of no discrimination (AUC = 0.5), indicating chance-level performance. UPDRS, Unified Parkinson’s Disease Rating Scale; FW, Forward walking; LANK, Less affected side of ankle; MaxJerk, Maximum jerk.

ROC analysis demonstrated that individual clinical and gait-derived features show varying levels of discriminative ability for distinguishing low-MoCA status. Among single features, UPDRS Part III and FW_LANK_MaxJerk exhibited relatively higher ROC–AUC values, whereas several gait-derived features showed ROC–AUC values close to or below 0.5, indicating limited discriminative ability when considered individually (Figure 3).

## 4. Discussion

This study systematically examined the relationships between motor-related features and cognitive performance in individuals with PD using an integrated framework combining regression analyses, network-based visualization, and ML-based classification. By comprehensively screening demographic and clinical characteristics, physical function, lifestyle factors, and gait parameters while deliberately excluding direct cognitive test scores, we sought to delineate motor–cognitive associations independent of conventional cognitive screening tools. Our findings demonstrate that global motor severity, balance dysfunction, and specific gait characteristics are meaningfully associated with cognitive status in PD and enable moderate discrimination of current cognitive status (low-MoCA). Importantly, these results suggest that routinely acquired motor examination measures and objective gait metrics can serve as practical, noninvasive adjunctive markers for identifying low-MoCA status in clinical settings. From a clinical perspective, this motor-based approach may facilitate earlier recognition of low-MoCA status, support risk stratification in routine neurological assessments, and inform timely referral for comprehensive cognitive evaluation and targeted intervention.

### 4.1. Motor and Clinical Correlates of Cognitive Performance

In the multivariable stepwise regression analysis, UPDRS Part III, turning-related stride length asymmetry (TurnPS_IMA_SLM), and ankle-level movement smoothness during forward walking (FW_LANK_MaxJerk) were independently associated with MoCA scores after adjustment for age, height, and BMI. The final model explained approximately 34.7% of the variance in current cognitive status (MoCA score), indicating that motor features capture a substantial, though partial, component of cognitive variability in PD.

UPDRS Part III, which evaluates motor symptom severity, emerged as the second most influential predictor, aligning with previous studies that link motor dysfunction—particularly bradykinesia, postural instability, and axial impairment—to low-MoCA [38]. These findings reinforce the role of overlapping motor–cognitive neural circuits, particularly within the basal ganglia and prefrontal cortex [2,39,40]. Consequently, axial and postural impairments captured by UPDRS Part III may indicate early cognitive vulnerability, underscoring its utility in integrated motor–cognitive assessments [41].

### 4.2. Gait-Derived Features as Digital Motor Markers

Among gait-derived features, TurnPS_IMA_SLM and FW_LANK_MaxJerk consistently emerged as relevant features across regression, network-based visualization, and classification analyses. These features capture complementary dimensions of gait control associated with cognitive processes, including spatial coordination during turning and movement smoothness during forward walking [42,43,44].

TurnPS_IMA_SLM reflects the SLM during preferred-speed 360° turning toward the inner step—a task that imposes substantial demands on spatial coordination, anticipatory postural adjustment, and executive control [38,41,42]. Turning-related gait alterations are recognized as particularly sensitive to early cognitive impairment in PD, as they require greater attentional resources and motor planning than straight walking [42,44,45]. FW_LANK_MaxJerk represents movement smoothness at the ankle of the less affected side during forward walking and may reflect compensatory or adaptive motor control strategies [46,47]. Increased jerk has been associated with impaired neuromuscular coordination and reduced movement efficiency—processes closely linked to executive dysfunction and attentional deficits [43,48,49]. Together, these findings underscore the relevance of distal, task-specific gait features in capturing neuromechanical disturbances associated with cognitive vulnerability in PD.

Importantly, the task- and side-specific nature of these gait markers highlights the role of motor asymmetry in PD [46,50]. Turning toward the more affected side imposes increased stabilization and coordination demands, while ankle-level smoothness on the less affected side may reflect compensatory control mechanisms during steady-state walking [42,43]. Such asymmetries provide physiologically meaningful insight into the manifestation of cognitive impairment within the motor system [42,43,51]. These findings are consistent with the classification results, in which these features were repeatedly selected as part of the final digital biomarker set.

### 4.3. Network-Based Interpretation of Motor–Cognitive Relationships

Network-based visualization provided complementary insight into the relational structure among cognitive, clinical, and motor features identified in the regression analysis. UPDRS Part III exhibited the strongest direct connection with MoCA, consistent with its prominent role in the regression model. TurnPS_IMA_SLM also showed a direct association with MoCA, reinforcing its contribution as an independent spatial gait marker reflecting coordination demands during directional turning [52,53].

Notably, FW_LANK_MaxJerk emerged as a functionally integrative node linking UPDRS Part III and MoCA, suggesting that ankle-level movement smoothness may reflect interactions between global motor severity and cognitive performance. Importantly, these network findings should be interpreted as exploratory and complementary to the regression results, providing a visual representation of inter-feature relationships rather than independent inferential evidence.

### 4.4. Classification of Low-MoCA Status

The classification models demonstrated moderate discriminative performance in distinguishing individuals with low-MoCA status. Logistic regression achieved stable and balanced performance, whereas the SVM with an RBF kernel demonstrated the highest overall classification performance. Despite the slightly higher performance of nonlinear models, logistic regression was selected as the primary model due to its interpretability and clinical transparency, which are essential for defining digital biomarkers. XGBoost, evaluated as a nonlinear benchmark within the same nested cross-validation framework, demonstrated comparable performance but did not provide a meaningful advantage over the other models. This finding suggests that increased model complexity did not substantially improve predictive performance in this dataset, likely due to the modest sample size and the relatively limited signal-to-noise ratio of the candidate features.

Importantly, comparative analyses showed that the clinical-only model already demonstrated strong discriminative performance, whereas the combined model achieved comparable performance. This suggests that while gait-derived features may not substantially improve overall classification accuracy, they provide complementary information reflecting distinct motor–cognitive characteristics not fully captured by conventional clinical measures. This finding reinforces the interpretation of gait-derived features as complementary rather than replacement markers.

These classification results were obtained without the inclusion of direct cognitive test scores, indicating that motor examination measures, physical function assessments, and gait-derived features alone encode meaningful information related to cognitive status in PD [52,54]. The RFECV-selected feature set—comprising UPDRS Part III, Mini-BEST, and three gait features—enabled characterization of cognitive–motor interactions, particularly reflecting deficits in executive control, balance regulation, and movement smoothness [55,56,57]. In addition, Mini-BEST—a standardized balance assessment—demonstrated notable predictive importance. This finding supports previous reports linking postural control impairments to cognitive dysfunction in PD [20]. Given that Mini-BEST evaluates multi-domain balance control (e.g., anticipatory postural adjustments, reactive postural control, and sensory orientation), its inclusion as a meaningful predictor aligns with the observed association between axial motor deficits and cognitive vulnerability [58]. The incorporation of Mini-BEST highlights the relevance of multi-system motor assessments for capturing subtle cognitive changes in PD.

Among the evaluated classifiers, logistic regression demonstrated robust and interpretable performance, supporting its potential applicability in clinical workflows [52,56]. While the proposed models are not intended to replace formal cognitive assessments, they may serve as complementary adjunctive tools to support clinical assessment in settings where cognitive testing is limited, impractical, or time-constrained [56]. Collectively, these findings underscore the clinical relevance of integrating quantitative gait-derived metrics and physical function measures to support the identification of cognitive vulnerability in PD.

### 4.5. Clinical Implications and Limitations

The findings suggest that integrating clinical characteristics with gait-derived features may provide complementary information for characterizing cognitive impairment in individuals with PD. The models are interpretable and computationally efficient; however, given the controlled laboratory setting and multimodal acquisition protocol, these findings should be interpreted as proof-of-concept rather than evidence of immediate clinical deployment. In addition, although nonlinear models demonstrated slightly higher classification performance, logistic regression was selected as the primary model due to its superior interpretability and clinical transparency, which are essential for defining digital biomarkers. This highlights an inherent trade-off between predictive performance and clinical applicability. The incorporation of continuous, noninvasive gait monitoring provides complementary value to traditional cognitive screening tools, supporting risk stratification, individualized therapeutic planning, and longitudinal monitoring.

Potential confounding factors should be considered when interpreting the present findings. Age was included as a covariate in the regression analyses, thereby partially accounting for its influence on both motor and cognitive function. Disease duration was also examined descriptively and did not differ significantly between groups, suggesting that the observed associations were not solely driven by disease progression. In addition, major neurological, orthopedic, and psychiatric comorbidities were excluded at the study design stage to minimize their potential impact on gait and cognitive measures. Nevertheless, residual confounding cannot be completely ruled out, and future studies incorporating more comprehensive clinical profiling and larger cohorts are warranted to further clarify these relationships.

Nevertheless, several limitations of this study should be acknowledged. First, the relatively small sample size may limit the generalizability of the findings, underscoring the need for future validation in larger, more diverse, and multicenter cohorts. Second, the cross-sectional study design precludes any inference regarding the temporal progression of cognitive decline. Although the identified motor-derived biomarkers were associated with current cognitive status, it remains unclear whether they can predict future cognitive deterioration. Longitudinal studies are therefore required to validate the prognostic value of these biomarkers. Third, because the data collection occurred exclusively during patients’ “ON” medication states, the motor and cognitive assessments may not fully disentangle intrinsic disease-related motor–cognitive interactions from medication-induced effects. Given that dopaminergic medication can influence both motor and cognitive domains, one concern is that medication dosage may confound the interpretation of clinical features. However, in our cohort, all participants were receiving dopaminergic therapy and were assessed in the ON state. Spearman correlation analysis revealed no significant association between LEDD and MoCA score (ρ = −0.066, *p* = 0.510), suggesting that cognitive variability was not strongly associated with dopaminergic dosage in this cohort, although medication-related effects cannot be fully excluded. Future studies should evaluate performance in both “ON” and “OFF” states to enable more comprehensive cognitive–motor profiling. Fourth, as gait data were collected under controlled laboratory conditions, the findings should be interpreted as proof-of-concept evidence derived under controlled laboratory conditions and may not fully represent real-world variability. Incorporating real-world gait monitoring could enhance ecological validity and strengthen clinical applicability. Finally, the absence of external validation represents a key limitation of the present study. To mitigate this limitation, stratified group-wise cross-validation was employed to ensure robust within-cohort generalization while preserving subject-level independence. Although this approach does not fully replace validation using an independent external dataset, it provides a conservative internal evaluation of model performance within the current cohort by ensuring that data from the same individual is never included in both training and test sets. Compared with conventional random or stratified k-fold cross-validation, group-wise cross-validation better approximates real-world deployment scenarios and reduces the risk of information leakage. Future studies are required to validate the proposed models in independent cohorts and across multiple clinical sites to establish broader applicability and generalizability of the identified biomarkers.

## 5. Conclusions

This study demonstrates that motor-related features—including clinical motor severity, balance-related physical function, and task-specific gait-derived metrics—are meaningfully associated with cognitive status in individuals with PD and enable moderate discrimination of low-MoCA status without reliance on direct cognitive test scores. Using an integrated analytical framework combining regression analysis, network-based visualization, and ML-based classification, we identified UPDRS Part III, Mini-BEST, and distinct gait features related to turning performance and movement smoothness as key contributors to motor–cognitive associations. Although the proposed models are not intended to replace formal neuropsychological assessments, their interpretable structure and computational efficiency support their potential as adjunctive tools for characterizing cognitive status, although their clinical applicability requires further validation and resource-limited settings. Collectively, these findings suggest that motor- and gait-derived features may provide complementary information to conventional clinical assessments for characterizing cognitive status in PD. While the proposed models demonstrate moderate discriminative performance, their role should be considered adjunctive, and further validation in longitudinal and real-world settings is required. Future studies incorporating longitudinal follow-up, free-living gait data, and independent external validation are required to establish the predictive and clinical utility of these biomarkers.

## Figures and Tables

**Table 1:** Demographics and clinical characteristics of the study participants.

	Normal-MoCA Group (*n* = 60)	Low-MoCA Group (*n* = 42)	*p*-Value
	Mean ± SD		
Sex (male/female)	24/36	22/20	
Age (years)	65.83 ± 7.52	71.24 ± 5.36	**<0.001 ^a^**
Height (cm)	161.47 ± 8.16	158.79 ± 8.66	0.114 ^a^
Body weight (kg)	62.95 ± 11.89	62.82 ± 8.67	0.731 ^b^
BMI (kg/m^2^)	24.01 ± 3.29	24.89 ± 2.67	0.063 ^b^
Symptom duration (years)	5.44 ± 3.26	6.08 ± 5.58	0.600 ^b^
Treatment duration (years)	4.19 ± 3.18	5.06 ± 5.35	0.303 ^b^
LEDD (mg/day)	542.89 ± 316.50	549.12 ± 250.88	0.514 ^b^
Hoehn and Yahr scale (stages)	1.83 ± 0.69	2.14 ± 0.72	**0.032 ^b^**
UPDRS total (score)	47.78 ± 19.83	64.13 ± 24.40	**<0.001 ^a^**
UPDRS Part I (score)	9.53 ± 5.33	10.88 ± 5.00	0.180 ^b^
UPDRS Part II (score)	11.70 ± 6.38	14.60 ± 8.06	0.071 ^b^
UPDRS Part III (score)	24.62 ± 12.64	36.35 ± 16.10	**<0.001 ^b^**
UPDRS Part IV (score)	1.93 ± 2.73	1.40 ± 2.58	0.212 ^b^
MoCA (score)	27.83 ± 1.24	23.00 ± 2.36	**<0.001 ^b^**
Education (years)	12.73 ± 3.25	10.36 ± 3.62	**0.001 ^b^**

The data are presented as mean ± standard deviation. Statistically significant differences between groups are shown in bold (*p* < 0.05). BMI, Body mass index; LEDD, Levodopa-equivalent daily dose; UPDRS, Unified Parkinson’s Disease Rating Scale; MoCA, Montreal Cognitive Assessment. ^a^ Independent-samples *t*-test. ^b^ Mann–Whitney *U* test.

**Table 2:** Gait features derived from wearable sensors.

Feature	Mathematical Expression
$\mathrm{Maximum}\;\mathrm{jerk}\;(m/s^{3})$	$\frac{dAcceleration}{dt}$
$\mathrm{Maximum}\;\mathrm{angular}\;\mathrm{velocity}\;\mathrm{jerk}\;(rad/s^{3})$	$\frac{d^{2}AngularVelocity}{{dt}^{2}}$
$\mathrm{Mean}\;\mathrm{acceleration}\;(m/s^{2})$	$mean\left(\sqrt{{acc}_{x}^{2}+{acc}_{y}^{2}+{acc}_{z}^{2}}\right)$
Mean gyroscopes (rad/s)	$mean\left(\sqrt{{gyr}_{x}^{2}+{gyr}_{y}^{2}+{gyr}_{z}^{2}}\right)$
$\mathrm{Maximum}\;\mathrm{acceleration}\;(m/s^{2})$	$max\left(\sqrt{{acc}_{x}^{2}+{acc}_{y}^{2}+{acc}_{z}^{2}}\right)$
Maximum gyroscopes (rad/s)	$max\left(\sqrt{{gyr}_{x}^{2}+{gyr}_{y}^{2}+{gyr}_{z}^{2}}\right)$
$\mathrm{RMS}\;\mathrm{acceleration}\;(m/s^{2})$	$\sqrt{mean({acc}_{x}^{2}+{acc}_{y}^{2}+{acc}_{z}^{2})}$
RMS gyroscopes (rad/s)	$\sqrt{mean({gyr}_{x}^{2}+{gyr}_{y}^{2}+{gyr}_{z}^{2})}$
Sample entropy acceleration	$-\log\left(\frac{Aacc}{Bacc}\right)$
Sample entropy gyroscopes	$-\log\left(\frac{Agyr}{Bgyr}\right)$

Jerk was defined as the first derivative of linear acceleration with respect to time, and angular jerk as the second derivative of angular velocity. Sample entropy was computed using parameters m = 2 and r = 0.2 × SD of the signal. RMS, root mean square; acc, acceleration; gyr, gyroscope; *A*: Total number of similar vector pairs of length *m* + 1 (i.e., *m* + 1 consecutive data points) that remain within a defined tolerance *r*; *B*, total number of similar vector pairs of length *m* (i.e., *m* consecutive data points) within the same tolerance *r*.

**Table 3:** Stepwise multiple linear regression analysis predicting MoCA scores.

Feature	*β* (SE)	*t*	*p*-Value
UPDRS Part III	−0.252 (0.085)	−2.972	0.004
TurnPS_IMA_SLM	0.258 (0.085)	3.042	0.003
FW_LANK_MaxJerk	−0.180 (0.083)	−2.160	0.033

Adjusted R^2^ = 0.347. SE, Standard error; MoCA, Montreal Cognitive Assessment; UPDRS, Unified Parkinson’s Disease Rating Scale; TurnPS_IMA, 360° turns at preferred speeds in the direction of the inner step of the more affected side; SLM, Stride length of the more affected side; FW, Forward walking; LANK, Less affected side of ankle; MaxJerk, Maximum jerk; *p* < 0.05.

**Table 4:** Classification performance of machine learning models for identifying low-MoCA status using nested cross-validation.

Model	Accuracy (Mean ± SD)	ROC–AUC (Mean ± SD)	Sensitivity (95% CI)	Specificity (95% CI)	PPV (95% CI)	NPV (95% CI)
Logistic regression	0.658 ± 0.138	0.737 ± 0.194	0.524 (0.370–0.667)	0.750 (0.636–0.855)	0.595 (0.424–0.744)	0.692 (0.571–0.797)
SVM (linear kernel)	0.667 ± 0.122	0.761 ± 0.188	0.524 (0.372–0.659)	0.767 (0.655–0.869)	0.611 (0.444–0.763)	0.697 (0.587–0.806)
SVM (RBF kernel)	0.696 ± 0.127	0.780 ± 0.139	0.548 (0.400–0.686)	0.800 (0.698–0.895)	0.657 (0.488–0.805)	0.716 (0.606–0.818)
XGBoost	0.637 ± 0.140	0.740 ± 0.162	0.524 (0.367–0.667)	0.717 (0.600–0.825)	0.564 (0.400–0.714)	0.683 (0.561–0.797)

The final feature panel included UPDRS Part III, Mini-BEST, FW_LANK_MaxJerk, TurnFS_IMA_SLM, and TurnPS_IMA_SLL. Confidence intervals for sensitivity, specificity, positive predictive value (PPV), and negative predictive value (NPV) were estimated using bootstrap resampling. MoCA, Montreal Cognitive Assessment; SVM, Support vector machine; RBF, Radial basis function; ROC-AUC, Area under the receiver operating characteristic curve.

## Data Availability

The datasets supporting the findings of this study are available from the corresponding author upon reasonable request. No open-source code is available for this study. All data preprocessing, modeling, and analyses were performed in a conda-managed Python environment (Python v3.10.18). Statistical analysis and machine learning procedures were implemented using scikit-learn (v1.1.3). Data handling and feature processing were conducted using pandas (v2.2.3), NumPy (v1.26.4), and SciPy (v1.15.3). Network-based visualization was implemented using NetworkX (v3.4.2), and figures were generated using Matplotlib (v3.10.0) and Seaborn (v0.13.2). Excel file input/output was managed using Openpyxl (v3.1.5). The analysis code is not publicly available but may be obtained from the corresponding author upon reasonable request.

## Supplementary Materials

The following supporting information can be downloaded at: https://www.mdpi.com/article/10.3390/s26082503/s1, Supplementary Table S1. Summary of multi-feature used in the analysis, including clinical, physical function, lifestyle, and gait-derived features; Supplementary Table S2. Univariate regression analysis of multi-features associated with MoCA scores; Supplementary Table S3. Baseline comparison of clinical-only and gait/sensor-only models for evaluating the incremental value of sensor-derived features in classifying low-MoCA status.

## Abbreviations

The following abbreviations are used in this manuscript:

BMI	body mass index
FW	forward walking
LANK	less affected side of ankle
LASSO	least absolute shrinkage and selection operator
LEDD	levodopa-equivalent daily dose
Mini-BEST	Mini-Balance Evaluation Systems Test
ML	machine learning
MoCA	Montreal Cognitive Assessment
NPV	Negative predictive value
PD	Parkinson’s disease
PPV	Positive predictive value
RBF	radial basis function
RFE	recursive feature elimination
RFECV	recursive feature elimination with cross-validation
RMS	root-mean-square
ROC	receiver operating characteristic
ROC-AUC	area under the receiver operating characteristic curve
SD	standard deviation
SLL	stride length of the less affected side
SLM	stride length of the more affected side
SVM	support vector machine
TurnFS_IMA	360° turns at maximum speeds in the direction of the inner step of the more affected side
TurnPS_IMA	360° turns at preferred speeds in the direction of the inner step of the more affected side
UPDRS	Unified Parkinson’s Disease Rating Scale
VIF	variance inflation factor

## References

[1] Leroi I., McDonald K., Pantula H., Harbishettar V. (2012). Cognitive impairment in Parkinson disease: Impact on quality of life, disability, and caregiver burden. J. Geriatr. Psychiatry Neurol..

[2] Sasikumar S., Strafella A.P. (2020). Imaging mild cognitive impairment and dementia in Parkinson’s disease. Front. Neurol..

[3] Aarsland D., Bronnick K., Williams-Gray C., Weintraub D., Marder K., Kulisevsky J., Burn D., Barone P., Pagonabarraga J., Allcock L. (2010). Mild cognitive impairment in Parkinson disease: A multicenter pooled analysis. Neurology.

[4] Palavra N.C., Naismith S.L., Lewis S.J. (2013). Mild cognitive impairment in Parkinson’s disease: A review of current concepts. Neurol. Res. Int..

[5] Salmanpour M.R., Shamsaei M., Saberi A., Setayeshi S., Klyuzhin I.S., Sossi V., Rahmim A. (2019). Optimized machine learning methods for prediction of cognitive outcome in Parkinson’s disease. Comput. Biol. Med..

[6] Harvey J., Reijnders R.A., Cavill R., Duits A., Köhler S., Eijssen L., Rutten B.P., Shireby G., Torkamani A., Creese B. (2022). Machine learning-based prediction of cognitive outcomes in de novo Parkinson’s disease. npj Park. Dis..

[7] Booth S., Park K.W., Lee C.S., Ko J.H. (2022). Predicting cognitive decline in Parkinson’s disease using FDG-PET-based supervised learning. J. Clin. Investig..

[8] Gramotnev G., Gramotnev D.K., Gramotnev A. (2019). Parkinson’s disease prognostic scores for progression of cognitive decline. Sci. Rep..

[9] Hayete B., Wuest D., Laramie J., McDonagh P., Church B., Eberly S., Lang A., Marek K., Runge K., Shoulson I. (2017). A Bayesian mathematical model of motor and cognitive outcomes in Parkinson’s disease. PLoS ONE.

[10] Hobson P., Meara J. (2015). Mild cognitive impairment in Parkinson’s disease and its progression onto dementia: A 16-year outcome evaluation of the Denbighshire cohort. Int. J. Geriatr. Psychiatry.

[11] Pigott K., Rick J., Xie S.X., Hurtig H., Chen-Plotkin A., Duda J.E., Morley J.F., Chahine L.M., Dahodwala N., Akhtar R.S. (2015). Longitudinal study of normal cognition in Parkinson disease. Neurology.

[12] Wood K.L., Myall D.J., Livingston L., Melzer T.R., Pitcher T.L., MacAskill M.R., Geurtsen G.J., Anderson T.J., Dalrymple-Alford J.C. (2016). Different PD-MCI criteria and risk of dementia in Parkinson’s disease: 4-year longitudinal study. npj Park. Dis..

[13] Gorji A., Jouzdani A.F. (2024). Machine learning for predicting cognitive decline within five years in Parkinson’s disease: Comparing cognitive assessment scales with DAT SPECT and clinical biomarkers. PLoS ONE.

[14] Kim H.M., Nazor C., Zabetian C.P., Quinn J.F., Chung K.A., Hiller A.L., Hu S., Leverenz J.B., Montine T.J., Edwards K.L. (2019). Prediction of cognitive progression in Parkinson’s disease using three cognitive screening measures. Clin. Park Relat. Disord..

[15] Almgren H., Camacho M., Hanganu A., Kibreab M., Camicioli R., Ismail Z., Forkert N.D., Monchi O. (2023). Machine learning-based prediction of longitudinal cognitive decline in early Parkinson’s disease using multimodal features. Sci. Rep..

[16] Camargo C.H., Tolentino E.S., Bronzini A., Ladeira M.A., Lima R., Schultz-Pereira G.L., Young-Blood M.R. (2016). Comparison of the use of screening tools for evaluating cognitive impairment in patients with Parkinson’s disease. Dement. Neuropsychol..

[17] Nasreddine Z.S., Phillips N.A., Bédirian V., Charbonneau S., Whitehead V., Collin I., Cummings J.L., Chertkow H. (2005). The Montreal Cognitive Assessment, MoCA: A brief screening tool for mild cognitive impairment. J. Am. Geriatr. Soc..

[18] Goldman J.G., Vernaleo B.A., Camicioli R., Dahodwala N., Dobkin R.D., Ellis T., Galvin J.E., Marras C., Edwards J., Fields J. (2018). Cognitive impairment in Parkinson’s disease: A report from a multidisciplinary symposium on unmet needs and future directions to maintain cognitive health. npj Park. Dis..

[19] Redgrave P., Rodriguez M., Smith Y., Rodriguez-Oroz M.C., Lehericy S., Bergman H., Agid Y., DeLong M.R., Obeso J.A. (2010). Goal-directed and habitual control in the basal ganglia: Implications for Parkinson’s disease. Nat. Rev. Neurosci..

[20] Barbosa A.F., Chen J., Freitag F., Valente D., De Oliveira Souza C., Voos M.C., Chien H.F. (2016). Gait, posture and cognition in Parkinson’s disease. Dement. Neuropsychol..

[21] Pieruccini-Faria F., Black S.E., Masellis M., Smith E.E., Almeida Q.J., Li K.Z.H., Bherer L., Camicioli R., Montero-Odasso M. (2021). Gait variability across neurodegenerative and cognitive disorders: Results from the Canadian Consortium of Neurodegeneration in Aging (CCNA) and the Gait and Brain Study. Alzheimers Dement..

[22] Coates L., Shi J., Rochester L., Del Din S., Pantall A. (2020). Entropy of real-world gait in Parkinson’s disease determined from wearable sensors as a digital marker of altered ambulatory behavior. Sensors.

[23] Shah V.V., McNames J., Mancini M., Carlson-Kuhta P., Nutt J.G., El-Gohary M., Lapidus J.A., Horak F.B., Curtze C. (2020). Digital biomarkers of mobility in Parkinson’s disease during daily living. J. Park. Dis..

[24] Zhang W., Ling Y., Chen Z., Ren K., Chen S., Huang P., Tan Y. (2024). Wearable sensor-based quantitative gait analysis in Parkinson’s disease patients with different motor subtypes. npj Digit. Med..

[25] Del Din S., Godfrey A., Mazzà C., Lord S., Rochester L. (2016). Free-living monitoring of Parkinson’s disease: Lessons from the field. Mov. Disord..

[26] Morris R., Martini D.N., Ramsey K., Kelly V.E., Smulders K., Hiller A., Chung K.A., Hu S.C., Zabetian C.P., Poston K.L. (2022). Cognition as a mediator for gait and balance impairments in GBA-related Parkinson’s disease. npj Park. Dis..

[27] Sun Y.M., Wang Z.Y., Liang Y.Y., Hao C.W., Shi C.H. (2024). Digital biomarkers for precision diagnosis and monitoring in Parkinson’s disease. npj Digit. Med..

[28] Albrecht F., Poulakis K., Freidle M., Johansson H., Ekman U., Volpe G., Westman E., Pereira J.B., Franzén E. (2022). Unraveling Parkinson’s disease heterogeneity using subtypes based on multimodal data. Park. Relat. Disord..

[29] Inguanzo A., Mohanty R., Poulakis K., Ferreira D., Segura B., Albrecht F., Muehlboeck J.S., Granberg T., Sjöström H., Svenningsson P. (2024). MRI subtypes in Parkinson’s disease across diverse populations and clustering approaches. npj Park. Dis..

[30] Amboni M., Ricciardi C., Adamo S., Nicolai E., Volzone A., Erro R., Cuoco S., Cesarelli G., Basso L., D’Addio G. (2022). Machine learning can predict mild cognitive impairment in Parkinson’s disease. Front Neurol..

[31] Russo M., Amboni M., Volzone A., Cuoco S., Camicioli R., Di Filippo F., Barone P., Romano M., Amato F., Ricciardi C. (2024). Kinematic and kinetic gait features associated with mild cognitive impairment in Parkinson’s disease. IEEE Trans. Neural Syst. Rehabil. Eng..

[32] Ghosh D., Pal S., Lutz M., Luo S. (2025). Ensemble survival analysis for preclinical cognitive decline prediction in Alzheimer’s disease using longitudinal biomarkers. arXiv.

[33] Janssen Daalen J.M., van den Bergh R., Prins E.M., Moghadam M.S.C., van den Heuvel R., Veen J., Mathur S., Meijerink H., Mirelman A., Darweesh S.K. (2024). Digital biomarkers for non-motor symptoms in Parkinson’s disease: The state of the art. npj Digit. Med..

[34] Longo C., Romano D.L., Malaguti M.C., Bacchin R., Papagno C. (2024). Cognitive reorganization in patients with Parkinson’s disease and Mild Cognitive Impairment: A neuropsychological network approach. Sci. Rep..

[35] Scharfenberg D., Kalbe E., Balzer-Geldsetzer M., Berg D., Hilker-Roggendorf R., Kassubek J., Liepelt-Scarfone I., Mollenhauer B., Reetz K., Riedel O. (2025). A network perspective on cognition in individuals with Parkinson’s disease. Alzheimers Dement..

[36] Spetsieris P.G., Eidelberg D. (2023). Parkinson’s disease progression: Increasing expression of an invariant common core subnetwork. NeuroImage Clin..

[37] Igual L., Seguí S. (2024). Network Analysis. Introduction to Data Science.

[38] Liampas I., Siokas V., Stamati P., Zoupa E., Tsouris Z., Provatas A., Kefalopoulou Z., Chroni E., Lyketsos C.G., Dardiotis E. (2025). Motor signs and incident dementia with Lewy bodies in older adults with mild cognitive impairment. J. Am. Geriatr. Soc..

[39] Di Tella S., Isernia S., Cabinio M., Rossetto F., Borgnis F., Pagliari C., Cazzoli M., Navarro J., Silveri M.C., Baglio F. (2024). Cognitive Reserve proxies can modulate motor and non-motor basal ganglia circuits in early Parkinson’s disease. Brain Imaging Behav..

[40] Sosnik R., Fahoum F., Katzir Z., Mirelman A., Maidan I. (2025). Key shifts in frontoparietal network activity in Parkinson’s disease. npj Park. Dis..

[41] Wilson H., Pagano G., Yousaf T., Polychronis S., De Micco R., Giordano B., Niccolini F., Politis M. (2020). Predict cognitive decline with clinical markers in Parkinson’s disease (PRECODE-1). J. Neural Transm..

[42] Burgos P.I., Silva-Batista C., Ragothaman A., Shah V.V., Carlson-Kuhta P., Horak F.B., Mancini M. (2025). Cognition is associated with daily-life mobility in people with Parkinson’s disease. Clin. Park Relat. Disord..

[43] Piet A., Geritz J., Garcia P., Irsfeld M., Li F., Huang X., Irshad M.T., Welzel J., Hansen C., Maetzler W. (2024). Predicting executive functioning from walking features in Parkinson’s disease using machine learning. Sci. Rep..

[44] Lander J.J., Moran M.F., Alexanian H.R. (2025). Correlations of gait kinematics and cognitive skills in Parkinson disease. PLoS ONE.

[45] Mohammadi H., Maghsoudpour A., Noroozian M., Mohammadian F. (2025). Talking during walking: The diagnostic potential of turn dynamics in Alzheimer’s disease, mild cognitive impairment and cognitive aging. Front. Aging Neurosci..

[46] Seuthe J., Hermanns H., Hulzinga F., D’Cruz N., Deuschl G., Ginis P., Nieuwboer A., Schlenstedt C. (2024). Gait asymmetry and symptom laterality in Parkinson’s disease: Two of a kind?. J. Neurol..

[47] Boonstra T.A., Schouten A.C., van Vugt J.P., Bloem B.R., van der Kooij H. (2014). Parkinson’s disease patients compensate for balance control asymmetry. J. Neurophysiol..

[48] Caronni A., Amadei M., Diana L., Sangalli G., Scarano S., Perucca L., Rota V., Bolognini N. (2025). In Parkinson’s disease, dual-tasking reduces gait smoothness during the straight-walking and turning-while-walking phases of the Timed Up and Go test. BMC Sports Sci. Med. Rehabil..

[49] Kazemi D., Chadeganipour A.S., Dehghani M., Ghorbali F. (2024). Associations of dual-task walking costs with cognition in Parkinson’s disease. Gait Posture.

[50] Barbieri F.A., de Campos D.D.S.F., Fukuchi C.A., Cupertino L., Pellegrino N.M., Angeles E.L., Coelho D.B. (2025). Inter-limb gait asymmetry in people with Parkinson’s disease. Hum. Mov. Sci..

[51] Amara A.W., Wood K.H., Miften A.M., Kleinschmidt L., White C.S., Joop A., Memon R.A., Pilkington J., Kongsuk J., Catiul C. (2025). Gait and balance dysfunction are associated with cognitive performance only in men with Parkinson’s disease. Clin. Park Relat. Disord..

[52] Morris R., Martini D.N., Smulders K., Kelly V.E., Zabetian C.P., Poston K., Hiller A., Chung K.A., Yang L., Hu S.C. (2019). Cognitive associations with comprehensive gait and static balance measures in Parkinson’s disease. Park. Relat. Disord..

[53] Pal G., O’Keefe J., Robertson-Dick E., Bernard B., Anderson S., Hall D. (2016). Global cognitive function and processing speed are associated with gait and balance dysfunction in Parkinson’s disease. J. Neuroeng. Rehabil..

[54] Lord S., Galna B., Coleman S., Yarnall A., Burn D., Rochester L. (2014). Cognition and gait show a selective pattern of association dominated by phenotype in incident Parkinson’s disease. Front. Aging Neurosci..

[55] Mirelman A., Bonato P., Camicioli R., Ellis T.D., Giladi N., Hamilton J.L., Hass C.J., Hausdorff J.M., Pelosin E., Almeida Q.J. (2019). Gait impairments in Parkinson’s disease. Lancet Neurol..

[56] Amboni M., Barone P., Hausdorff J.M. (2013). Cognitive contributions to gait and falls: Evidence and implications. Mov. Disord..

[57] Maidan I., Nieuwhof F., Bernad-Elazari H., Reelick M.F., Bloem B.R., Giladi N., Deutsch J.E., Hausdorff J.M., Claassen J.A., Mirelman A. (2016). The role of the frontal lobe in complex walking among patients with Parkinson’s disease and healthy older adults: An fNIRS study. Neurorehabilit. Neural Repair..

[58] Löfgren N., Lenholm E., Conradsson D., Ståhle A., Franzén E. (2014). The Mini-BESTest-a clinically reproducible tool for balance evaluations in mild to moderate Parkinson’s disease?. BMC Neurol..

