# Supplementary material for: Motor-Derived Digital Biomarkers for Identifying Low-MoCA Status in People with Parkinson’s Disease
Source: Sensors (Basel). 2026 Apr 18;26(8):2503. doi: 10.3390/s26082503 (PMC13119813; doi:10.3390/s26082503)
Supplement: Supplementary file 1 [file sensors-26-02503-s001.zip › Supplementary Table S1.pdf]

Supplementary Table S1. Summary of multi-feature used in the analysis, including clinical, physical function, lifestyle, and gait-derived features

Feature	Normal-MoCA group (Mean ± SD)	Low-MoCA group (Mean ± SD)	Test	p-value
Beck depression inventory	14.83 ± 8.14	14.05 ± 10.24	Mann–Whitney	0.434
Beck anxiety inventory	9.78 ± 8.17	9.29 ± 6.90	Mann–Whitney	0.889
PD sleep scale-2	15.35 ± 10.85	15.50 ± 10.67	Mann–Whitney	0.830
Epworth sleepiness scale	6.38 ± 4.68	5.81 ± 3.41	Mann–Whitney	0.814
Fatigue severity scale	33.23 ± 14.11	30.88 ± 14.64	t-test	0.420
Fall history	1.83 ± 0.38	1.69 ± 0.52	Mann–Whitney	0.141
Falls efficacy scale	24.77 ± 9.74	26.93 ± 12.15	Mann–Whitney	0.337
Self-efficacy for exercise scale;	62.75 ± 20.88	61.21 ± 23.85	Mann–Whitney	0.951
New freezing of gait questionnaire;	3.55 ± 7.01	4.10 ± 7.40	Mann–Whitney	0.684
Non-motor symptoms scale	42.00 ± 24.50	47.31 ± 26.91	Mann–Whitney	0.287
PD questionnaire-39	33.45 ± 18.50	36.24 ± 18.38	Mann–Whitney	0.409
Grip strength	28.05 ± 8.30	27.22 ± 7.71	t-test	0.605
Five-times sit-to-stand test	8.76 ± 2.38	10.45 ± 5.54	Mann–Whitney	0.108
Six-minute walk test	407.35 ± 95.70	364.17 ± 103.39	Mann–Whitney	**0.022**
Short physical performance battery	11.33 ± 1.20	10.26 ± 2.32	Mann–Whitney	**0.008**
Mini-balance evaluation systems test	24.12 ± 2.74	21.43 ± 3.83	Mann–Whitney	**0.000**
Nutrition quotient	66.10 ± 12.45	65.80 ± 8.83	t-test	0.887
36-item Short-Form Health Survey_physical	57.61 ± 20.28	58.35 ± 21.19	t-test	0.860
36-item Short-Form Health Survey_mental	64.89 ± 16.89	65.46 ± 17.29	Mann–Whitney	0.903
36-item Short-Form Health Survey_total	56.60 ± 20.40	59.52 ± 20.08	Mann–Whitney	0.543
International physical activity questionnaire	3271.38 ± 6097.55	2089.70 ± 2126.74	Mann–Whitney	0.420
TurnFS_IMA_WS	0.83 ± 0.15	0.71 ± 0.15	t-test	**0.000**
TurnFS_IMA_SLM	0.68 ± 0.10	0.61 ± 0.10	t-test	**0.001**

TurnFS_IMA_SLL	0.82 ± 0.13	0.78 ± 0.15	t-test	0.138
TurnFS_IMA_DSPM	34.55 ± 5.76	35.08 ± 6.07	Mann–Whitney	0.854
TurnFS_IMA_DSPL	34.06 ± 5.78	33.40 ± 6.03	t-test	0.582
TurnFS_IMA_CTM	0.38 ± 0.04	0.37 ± 0.05	Mann–Whitney	0.967
TurnFS_IMA_CTL	0.32 ± 0.03	0.35 ± 0.04	Mann–Whitney	**0.004**
TurnFS_OMA_WS	0.78 ± 0.14	0.68 ± 0.15	Mann–Whitney	**0.001**
TurnFS_OMA_SLM	0.81 ± 0.11	0.75 ± 0.14	t-test	**0.019**
TurnFS_OMA_SLL	0.64 ± 0.08	0.57 ± 0.12	Mann–Whitney	**0.000**
TurnFS_OMA_DSPM	34.96 ± 5.09	34.18 ± 6.24	Mann–Whitney	0.245
TurnFS_OMA_DSPL	36.16 ± 5.21	36.20 ± 6.22	t-test	0.968
TurnFS_OMA_CTM	0.33 ± 0.03	0.35 ± 0.03	Mann–Whitney	**0.008**
TurnFS_OMA_CTL	0.36 ± 0.04	0.37 ± 0.04	Mann–Whitney	0.064
TurnPS_IMA_WS	0.67 ± 0.12	0.60 ± 0.12	Mann–Whitney	**0.005**
TurnPS_IMA_SLM	0.64 ± 0.11	0.57 ± 0.10	Mann–Whitney	**0.004**
TurnPS_IMA_SLL	0.78 ± 0.12	0.72 ± 0.14	t-test	**0.015**
TurnPS_IMA_DSPM	39.28 ± 5.01	39.64 ± 6.43	t-test	0.764
TurnPS_IMA_DSPL	38.84 ± 5.34	38.12 ± 6.92	t-test	0.572
TurnPS_IMA_CTM	0.35 ± 0.04	0.36 ± 0.04	Mann–Whitney	0.336
TurnPS_IMA_CTL	0.34 ± 0.03	0.36 ± 0.04	t-test	**0.036**
TurnPS_OMA_WS	0.68 ± 0.12	0.62 ± 0.12	Mann–Whitney	**0.015**
TurnPS_OMA_SLM	0.76 ± 0.09	0.73 ± 0.13	t-test	0.173
TurnPS_OMA_SLL	0.63 ± 0.10	0.58 ± 0.11	t-test	**0.019**
TurnPS_OMA_DSPM	37.68 ± 4.91	37.75 ± 6.31	t-test	0.952
TurnPS_OMA_DSPL	38.77 ± 5.14	39.24 ± 6.35	Mann–Whitney	0.734
TurnPS_OMA_CTM	0.34 ± 0.03	0.35 ± 0.05	Mann–Whitney	0.241

TurnPS_OMA_CTL	0.36 ± 0.04	0.36 ± 0.05	Mann–Whitney	0.828
FW_WS	1.00 ± 0.20	0.89 ± 0.18	Mann–Whitney	**0.018**
FW_SLM	1.05 ± 0.15	0.97 ± 0.18	Mann–Whitney	0.078
FW_SLL	1.06 ± 0.15	0.97 ± 0.18	Mann–Whitney	**0.034**
FW_DSPM	25.57 ± 4.81	26.66 ± 6.96	Mann–Whitney	0.416
FW_DSPL	25.44 ± 5.00	26.95 ± 6.75	Mann–Whitney	0.162
BW_WS	0.52 ± 0.19	0.43 ± 0.18	t-test	**0.025**
BW_SLM	0.57 ± 0.19	0.49 ± 0.19	t-test	**0.029**
BW_SLL	0.56 ± 0.19	0.49 ± 0.19	t-test	0.065
BW_DSPM	37.29 ± 30.06	29.62 ± 24.85	Mann–Whitney	0.329
BW_DSPL	37.48 ± 29.38	29.13 ± 24.66	Mann–Whitney	0.217
TurnFS_IMA_MELB_Maxjerk	0.03 ± 0.01	0.03 ± 0.01	t-test	0.127
TurnFS_IMA_MELB_MaxAngVelJerk	0.42 ± 0.09	0.39 ± 0.12	Mann–Whitney	0.105
TurnFS_IMA_MELB_MeanAcc	10.19 ± 0.21	10.14 ± 0.14	Mann–Whitney	0.191
TurnFS_IMA_MELB_MaxAcc	16.14 ± 1.32	15.74 ± 1.68	Mann–Whitney	0.132
TurnFS_IMA_MELB_MeanGyr	98.09 ± 26.58	88.83 ± 25.00	t-test	0.076
TurnFS_IMA_MELB_MaxGyr	241.58 ± 62.50	217.07 ± 53.15	Mann–Whitney	0.089
TurnFS_IMA_MELB_RMSAcc	10.33 ± 0.26	10.27 ± 0.20	Mann–Whitney	0.191
TurnFS_IMA_MELB_RMSGyr	109.30 ± 28.28	98.63 ± 26.66	t-test	0.056
TurnFS_IMA_MELB_SampEnAcc	0.00 ± 0.01	0.00 ± 0.00	Mann–Whitney	0.590
TurnFS_IMA_MELB_SampEnGyr	0.85 ± 0.13	0.85 ± 0.11	Mann–Whitney	0.589
TurnFS_IMA_LELB_Maxjerk	0.03 ± 0.01	0.03 ± 0.01	Mann–Whitney	0.701
TurnFS_IMA_LELB_MaxAngVelJerk	0.41 ± 0.10	0.41 ± 0.12	Mann–Whitney	0.873
TurnFS_IMA_LELB_MeanAcc	10.25 ± 0.23	10.21 ± 0.22	Mann–Whitney	0.486
TurnFS_IMA_LELB_MaxAcc	16.09 ± 1.62	15.93 ± 2.24	Mann–Whitney	0.293

TurnFS_IMA_LELB_MeanGyr	95.25 ± 20.79	89.34 ± 26.75	t-test	0.233
TurnFS_IMA_LELB_MaxGyr	262.70 ± 53.71	251.73 ± 67.69	t-test	0.385
TurnFS_IMA_LELB_RMSAcc	10.38 ± 0.27	10.33 ± 0.27	Mann–Whitney	0.461
TurnFS_IMA_LELB_RMSGyr	108.24 ± 22.20	101.63 ± 28.87	t-test	0.216
TurnFS_IMA_LELB_SampEnAcc	0.00 ± 0.00	0.00 ± 0.01	Mann–Whitney	0.409
TurnFS_IMA_LELB_SampEnGyr	0.82 ± 0.16	0.87 ± 0.14	Mann–Whitney	0.048
TurnFS_IMA_MANK_Maxjerk	0.21 ± 0.04	0.23 ± 0.05	t-test	0.157
TurnFS_IMA_MANK_MaxAngVelJerk	1.30 ± 0.49	1.64 ± 0.80	Mann–Whitney	**0.021**
TurnFS_IMA_MANK_MeanAcc	13.45 ± 1.22	12.92 ± 1.20	Mann–Whitney	0.079
TurnFS_IMA_MANK_MaxAcc	50.55 ± 10.24	49.95 ± 11.54	Mann–Whitney	0.767
TurnFS_IMA_MANK_MeanGyr	152.11 ± 26.92	144.28 ± 34.77	t-test	0.224
TurnFS_IMA_MANK_MaxGyr	520.43 ± 114.67	541.64 ± 176.00	Mann–Whitney	0.954
TurnFS_IMA_MANK_RMSAcc	14.94 ± 1.67	14.31 ± 1.70	Mann–Whitney	0.130
TurnFS_IMA_MANK_RMSGyr	193.50 ± 32.18	185.00 ± 42.05	t-test	0.274
TurnFS_IMA_MANK_SampEnAcc	0.03 ± 0.02	0.03 ± 0.01	Mann–Whitney	0.809
TurnFS_IMA_MANK_SampEnGyr	0.50 ± 0.13	0.50 ± 0.16	Mann–Whitney	0.691
TurnFS_IMA_LANK_Maxjerk	0.18 ± 0.04	0.19 ± 0.05	Mann–Whitney	0.306
TurnFS_IMA_LANK_MaxAngVelJerk	1.39 ± 0.75	1.42 ± 0.57	Mann–Whitney	0.233
TurnFS_IMA_LANK_MeanAcc	14.51 ± 1.54	13.62 ± 1.37	Mann–Whitney	**0.013**
TurnFS_IMA_LANK_MaxAcc	53.36 ± 11.78	48.31 ± 9.47	t-test	**0.019**
TurnFS_IMA_LANK_MeanGyr	164.28 ± 33.12	149.91 ± 32.26	Mann–Whitney	0.063
TurnFS_IMA_LANK_MaxGyr	594.40 ± 203.77	537.39 ± 154.12	Mann–Whitney	0.119
TurnFS_IMA_LANK_RMSAcc	16.18 ± 2.03	15.05 ± 1.78	Mann–Whitney	**0.019**
TurnFS_IMA_LANK_RMSGyr	208.55 ± 39.84	189.65 ± 38.17	Mann–Whitney	**0.041**
TurnFS_IMA_LANK_SampEnAcc	0.02 ± 0.02	0.02 ± 0.01	Mann–Whitney	0.579

TurnFS_IMA_LANK_SampEnGyr	0.52 ± 0.18	0.48 ± 0.12	Mann–Whitney	0.212
TurnFS_IMA_PSI_Maxjerk	0.07 ± 0.03	0.07 ± 0.03	Mann–Whitney	0.448
TurnFS_IMA_PSI_MaxAngVelJerk	0.60 ± 0.42	0.52 ± 0.38	Mann–Whitney	0.287
TurnFS_IMA_PSI_MeanAcc	10.10 ± 0.12	10.07 ± 0.12	t-test	0.144
TurnFS_IMA_PSI_MaxAcc	18.98 ± 2.86	18.52 ± 2.93	Mann–Whitney	0.598
TurnFS_IMA_PSI_MeanGyr	71.72 ± 15.60	63.53 ± 17.59	t-test	**0.018**
TurnFS_IMA_PSI_MaxGyr	186.01 ± 64.40	158.30 ± 46.72	Mann–Whitney	**0.009**
TurnFS_IMA_PSI_RMSAcc	10.28 ± 0.20	10.23 ± 0.20	Mann–Whitney	0.205
TurnFS_IMA_PSI_RMSGyr	83.83 ± 17.78	73.70 ± 19.58	t-test	**0.009**
TurnFS_IMA_PSI_SampEnAcc	0.02 ± 0.02	0.02 ± 0.01	Mann–Whitney	0.109
TurnFS_IMA_PSI_SampEnGyr	0.62 ± 0.09	0.62 ± 0.09	Mann–Whitney	0.905
TurnFS_IMA_T10_Maxjerk	0.06 ± 0.02	0.06 ± 0.02	Mann–Whitney	0.598
TurnFS_IMA_T10_MaxAngVelJerk	0.81 ± 0.40	0.89 ± 0.40	Mann–Whitney	0.186
TurnFS_IMA_T10_MeanAcc	10.01 ± 0.08	10.00 ± 0.06	Mann–Whitney	0.696
TurnFS_IMA_T10_MaxAcc	20.41 ± 2.45	20.49 ± 3.46	t-test	0.895
TurnFS_IMA_T10_MeanGyr	70.01 ± 14.87	63.08 ± 17.38	t-test	0.039
TurnFS_IMA_T10_MaxGyr	189.76 ± 47.12	180.65 ± 58.80	t-test	0.407
TurnFS_IMA_T10_RMSAcc	10.21 ± 0.15	10.19 ± 0.16	Mann–Whitney	0.579
TurnFS_IMA_T10_RMSGyr	82.21 ± 16.96	73.95 ± 19.66	t-test	0.030
TurnFS_IMA_T10_SampEnAcc	0.03 ± 0.02	0.04 ± 0.02	Mann–Whitney	0.120
TurnFS_IMA_T10_SampEnGyr	0.61 ± 0.09	0.62 ± 0.08	t-test	0.803
TurnFS_OMA_MELB_Maxjerk	0.03 ± 0.01	0.02 ± 0.01	t-test	**0.018**
TurnFS_OMA_MELB_MaxAngVelJerk	0.42 ± 0.10	0.38 ± 0.10	Mann–Whitney	**0.028**
TurnFS_OMA_MELB_MeanAcc	10.34 ± 0.33	10.24 ± 0.21	Mann–Whitney	0.074
TurnFS_OMA_MELB_MaxAcc	15.87 ± 1.59	15.52 ± 1.59	Mann–Whitney	0.252

TurnFS_OMA_MELB_MeanGyr	100.25 ± 25.88	90.62 ± 25.49	Mann–Whitney	0.109
TurnFS_OMA_MELB_MaxGyr	270.13 ± 70.60	232.73 ± 57.54	Mann–Whitney	**0.007**
TurnFS_OMA_MELB_RMSAcc	10.47 ± 0.38	10.36 ± 0.26	Mann–Whitney	0.071
TurnFS_OMA_MELB_RMSGyr	113.33 ± 28.07	101.30 ± 27.05	Mann–Whitney	0.063
TurnFS_OMA_MELB_SampEnAcc	0.00 ± 0.00	0.00 ± 0.00	Mann–Whitney	0.293
TurnFS_OMA_MELB_SampEnGyr	0.82 ± 0.14	0.81 ± 0.11	Mann–Whitney	0.637
TurnFS_OMA_LELB_Maxjerk	0.03 ± 0.01	0.03 ± 0.01	t-test	0.290
TurnFS_OMA_LELB_MaxAngVelJerk	0.42 ± 0.10	0.40 ± 0.11	t-test	0.358
TurnFS_OMA_LELB_MeanAcc	10.11 ± 0.12	10.10 ± 0.16	Mann–Whitney	0.556
TurnFS_OMA_LELB_MaxAcc	15.93 ± 1.45	15.60 ± 1.87	Mann–Whitney	0.132
TurnFS_OMA_LELB_MeanGyr	95.21 ± 17.07	86.89 ± 23.63	t-test	0.055
TurnFS_OMA_LELB_MaxGyr	239.32 ± 48.52	219.27 ± 59.41	t-test	0.075
TurnFS_OMA_LELB_RMSAcc	10.24 ± 0.16	10.22 ± 0.22	Mann–Whitney	0.420
TurnFS_OMA_LELB_RMSGyr	106.49 ± 18.63	97.16 ± 26.01	t-test	0.050
TurnFS_OMA_LELB_SampEnAcc	0.00 ± 0.00	0.00 ± 0.00	Mann–Whitney	0.281
TurnFS_OMA_LELB_SampEnGyr	0.84 ± 0.15	0.86 ± 0.15	Mann–Whitney	0.235
TurnFS_OMA_MANK_Maxjerk	0.18 ± 0.04	0.18 ± 0.04	Mann–Whitney	0.809
TurnFS_OMA_MANK_MaxAngVelJerk	1.17 ± 0.46	1.51 ± 0.72	Mann–Whitney	**0.003**
TurnFS_OMA_MANK_MeanAcc	14.31 ± 1.45	13.57 ± 1.54	Mann–Whitney	**0.025**
TurnFS_OMA_MANK_MaxAcc	51.15 ± 12.22	47.23 ± 9.95	Mann–Whitney	0.255
TurnFS_OMA_MANK_MeanGyr	160.49 ± 21.87	151.86 ± 36.77	t-test	0.178
TurnFS_OMA_MANK_MaxGyr	544.89 ± 92.56	557.84 ± 185.63	Mann–Whitney	0.716
TurnFS_OMA_MANK_RMSAcc	15.99 ± 1.96	15.02 ± 2.06	Mann–Whitney	**0.032**
TurnFS_OMA_MANK_RMSGyr	202.71 ± 26.52	192.70 ± 45.82	Mann–Whitney	0.136
TurnFS_OMA_MANK_SampEnAcc	0.03 ± 0.01	0.02 ± 0.01	Mann–Whitney	0.158

TurnFS_OMA_MANK_SampEnGyr	0.51 ± 0.12	0.51 ± 0.14	Mann–Whitney	0.452
TurnFS_OMA_LANK_Maxjerk	0.21 ± 0.05	0.22 ± 0.05	Mann–Whitney	0.255
TurnFS_OMA_LANK_MaxAngVelJerk	1.55 ± 0.92	1.58 ± 0.57	Mann–Whitney	0.084
TurnFS_OMA_LANK_MeanAcc	13.61 ± 1.20	12.81 ± 1.06	Mann–Whitney	**0.002**
TurnFS_OMA_LANK_MaxAcc	50.70 ± 10.96	47.78 ± 9.20	t-test	0.148
TurnFS_OMA_LANK_MeanGyr	160.53 ± 30.51	143.71 ± 31.27	Mann–Whitney	**0.028**
TurnFS_OMA_LANK_MaxGyr	572.95 ± 217.10	524.91 ± 138.28	Mann–Whitney	0.525
TurnFS_OMA_LANK_RMSAcc	15.07 ± 1.61	14.09 ± 1.43	Mann–Whitney	**0.008**
TurnFS_OMA_LANK_RMSGyr	202.84 ± 37.87	182.29 ± 37.14	Mann–Whitney	**0.026**
TurnFS_OMA_LANK_SampEnAcc	0.03 ± 0.01	0.03 ± 0.01	t-test	0.358
TurnFS_OMA_LANK_SampEnGyr	0.54 ± 0.18	0.46 ± 0.13	Mann–Whitney	**0.037**
TurnFS_OMA_PSI_Maxjerk	0.07 ± 0.02	0.07 ± 0.02	Mann–Whitney	0.252
TurnFS_OMA_PSI_MaxAngVelJerk	0.62 ± 0.47	0.48 ± 0.29	Mann–Whitney	0.127
TurnFS_OMA_PSI_MeanAcc	10.11 ± 0.12	10.06 ± 0.13	Mann–Whitney	0.089
TurnFS_OMA_PSI_MaxAcc	18.80 ± 2.74	18.34 ± 2.75	Mann–Whitney	0.584
TurnFS_OMA_PSI_MeanGyr	72.65 ± 13.19	63.44 ± 16.88	t-test	**0.004**
TurnFS_OMA_PSI_MaxGyr	188.02 ± 61.14	154.83 ± 40.54	Mann–Whitney	**0.001**
TurnFS_OMA_PSI_RMSAcc	10.28 ± 0.20	10.22 ± 0.20	Mann–Whitney	0.096
TurnFS_OMA_PSI_RMSGyr	84.80 ± 15.31	73.25 ± 18.93	t-test	**0.002**
TurnFS_OMA_PSI_SampEnAcc	0.02 ± 0.02	0.02 ± 0.01	Mann–Whitney	**0.041**
TurnFS_OMA_PSI_SampEnGyr	0.61 ± 0.10	0.60 ± 0.09	Mann–Whitney	0.538
TurnFS_OMA_T10_Maxjerk	0.07 ± 0.02	0.06 ± 0.02	Mann–Whitney	0.686
TurnFS_OMA_T10_MaxAngVelJerk	0.86 ± 0.47	0.88 ± 0.42	Mann–Whitney	0.452
TurnFS_OMA_T10_MeanAcc	10.02 ± 0.08	10.00 ± 0.07	Mann–Whitney	0.507
TurnFS_OMA_T10_MaxAcc	20.44 ± 2.34	20.11 ± 3.24	Mann–Whitney	0.473

TurnFS_OMA_T10_MeanGyr	71.32 ± 12.76	63.17 ± 16.78	t-test	**0.010**
TurnFS_OMA_T10_MaxGyr	196.98 ± 54.67	176.73 ± 62.04	Mann–Whitney	0.094
TurnFS_OMA_T10_RMSAcc	10.22 ± 0.16	10.18 ± 0.16	Mann–Whitney	0.284
TurnFS_OMA_T10_RMSGyr	83.79 ± 14.95	73.66 ± 19.26	t-test	**0.006**
TurnFS_OMA_T10_SampEnAcc	0.03 ± 0.02	0.03 ± 0.02	Mann–Whitney	0.215
TurnFS_OMA_T10_SampEnGyr	0.61 ± 0.09	0.60 ± 0.08	t-test	0.283
TurnPS_IMA_MELB_Maxjerk	0.03 ± 0.01	0.02 ± 0.01	Mann–Whitney	0.386
TurnPS_IMA_MELB_MaxAngVelJerk	0.34 ± 0.08	0.34 ± 0.09	Mann–Whitney	0.375
TurnPS_IMA_MELB_MeanAcc	10.09 ± 0.12	10.07 ± 0.10	Mann–Whitney	0.706
TurnPS_IMA_MELB_MaxAcc	15.29 ± 1.08	15.05 ± 1.31	Mann–Whitney	0.353
TurnPS_IMA_MELB_MeanGyr	81.56 ± 20.98	75.47 ± 20.55	t-test	0.147
TurnPS_IMA_MELB_MaxGyr	208.82 ± 54.02	195.38 ± 49.53	Mann–Whitney	0.284
TurnPS_IMA_MELB_RMSAcc	10.18 ± 0.14	10.16 ± 0.13	Mann–Whitney	0.409
TurnPS_IMA_MELB_RMSGyr	91.84 ± 22.77	84.50 ± 21.88	t-test	0.105
TurnPS_IMA_MELB_SampEnAcc	0.00 ± 0.00	0.00 ± 0.00	Mann–Whitney	0.671
TurnPS_IMA_MELB_SampEnGyr	0.83 ± 0.14	0.83 ± 0.11	Mann–Whitney	0.416
TurnPS_IMA_LELB_Maxjerk	0.02 ± 0.01	0.02 ± 0.01	Mann–Whitney	0.895
TurnPS_IMA_LELB_MaxAngVelJerk	0.34 ± 0.09	0.35 ± 0.09	Mann–Whitney	0.503
TurnPS_IMA_LELB_MeanAcc	10.07 ± 0.11	10.08 ± 0.17	Mann–Whitney	0.873
TurnPS_IMA_LELB_MaxAcc	15.04 ± 0.97	14.99 ± 1.48	t-test	0.834
TurnPS_IMA_LELB_MeanGyr	77.76 ± 17.55	74.71 ± 22.35	t-test	0.463
TurnPS_IMA_LELB_MaxGyr	221.43 ± 56.83	212.13 ± 54.83	Mann–Whitney	0.393
TurnPS_IMA_LELB_RMSAcc	10.16 ± 0.13	10.17 ± 0.21	Mann–Whitney	0.726
TurnPS_IMA_LELB_RMSGyr	89.02 ± 19.57	85.53 ± 24.44	t-test	0.444
TurnPS_IMA_LELB_SampEnAcc	0.00 ± 0.00	0.00 ± 0.00	Mann–Whitney	0.579

TurnPS_IMA_LELB_SampEnGyr	0.80 ± 0.14	0.86 ± 0.17	Mann–Whitney	**0.007**
TurnPS_IMA_MANK_Maxjerk	0.20 ± 0.05	0.22 ± 0.05	t-test	0.051
TurnPS_IMA_MANK_MaxAngVelJerk	1.26 ± 0.52	1.56 ± 0.73	Mann–Whitney	**0.011**
TurnPS_IMA_MANK_MeanAcc	12.27 ± 0.79	12.01 ± 0.75	Mann–Whitney	0.244
TurnPS_IMA_MANK_MaxAcc	41.57 ± 9.73	43.22 ± 8.25	Mann–Whitney	0.129
TurnPS_IMA_MANK_MeanGyr	130.21 ± 20.28	122.91 ± 24.30	t-test	0.115
TurnPS_IMA_MANK_MaxGyr	468.68 ± 113.33	464.85 ± 119.80	Mann–Whitney	0.900
TurnPS_IMA_MANK_RMSAcc	13.27 ± 1.10	13.02 ± 1.12	t-test	0.280
TurnPS_IMA_MANK_RMSGyr	167.44 ± 25.39	159.34 ± 29.35	t-test	0.151
TurnPS_IMA_MANK_SampEnAcc	0.02 ± 0.01	0.02 ± 0.01	Mann–Whitney	0.820
TurnPS_IMA_MANK_SampEnGyr	0.44 ± 0.11	0.43 ± 0.11	Mann–Whitney	0.681
TurnPS_IMA_LANK_Maxjerk	0.17 ± 0.04	0.19 ± 0.04	Mann–Whitney	**0.030**
TurnPS_IMA_LANK_MaxAngVelJerk	1.38 ± 0.73	1.43 ± 0.48	Mann–Whitney	0.112
TurnPS_IMA_LANK_MeanAcc	12.93 ± 0.89	12.48 ± 0.93	t-test	0.016
TurnPS_IMA_LANK_MaxAcc	41.51 ± 9.18	41.82 ± 8.58	Mann–Whitney	0.452
TurnPS_IMA_LANK_MeanGyr	140.31 ± 22.32	129.11 ± 25.74	t-test	**0.025**
TurnPS_IMA_LANK_MaxGyr	506.06 ± 164.51	456.84 ± 90.26	Mann–Whitney	0.166
TurnPS_IMA_LANK_RMSAcc	13.99 ± 1.20	13.47 ± 1.25	t-test	**0.040**
TurnPS_IMA_LANK_RMSGyr	178.78 ± 27.80	164.12 ± 30.37	t-test	**0.015**
TurnPS_IMA_LANK_SampEnAcc	0.01 ± 0.01	0.02 ± 0.01	Mann–Whitney	0.346
TurnPS_IMA_LANK_SampEnGyr	0.47 ± 0.14	0.43 ± 0.10	Mann–Whitney	0.143
TurnPS_IMA_PSI_Maxjerk	0.06 ± 0.02	0.06 ± 0.02	t-test	0.690
TurnPS_IMA_PSI_MaxAngVelJerk	0.46 ± 0.24	0.40 ± 0.20	Mann–Whitney	0.266
TurnPS_IMA_PSI_MeanAcc	10.00 ± 0.07	9.99 ± 0.08	t-test	0.283
TurnPS_IMA_PSI_MaxAcc	17.17 ± 1.76	16.92 ± 2.15	t-test	0.536

TurnPS_IMA_PSI_MeanGyr	58.01 ± 10.47	52.22 ± 12.88	t-test	**0.018**
TurnPS_IMA_PSI_MaxGyr	155.06 ± 37.94	131.19 ± 27.77	Mann–Whitney	**0.003**
TurnPS_IMA_PSI_RMSAcc	10.11 ± 0.11	10.09 ± 0.12	t-test	0.280
TurnPS_IMA_PSI_RMSGyr	68.81 ± 12.40	61.13 ± 14.52	t-test	**0.007**
TurnPS_IMA_PSI_SampEnAcc	0.02 ± 0.02	0.02 ± 0.01	Mann–Whitney	0.269
TurnPS_IMA_PSI_SampEnGyr	0.60 ± 0.08	0.61 ± 0.10	Mann–Whitney	0.494
TurnPS_IMA_T10_Maxjerk	0.05 ± 0.02	0.05 ± 0.02	Mann–Whitney	0.752
TurnPS_IMA_T10_MaxAngVelJerk	0.65 ± 0.33	0.71 ± 0.33	Mann–Whitney	0.246
TurnPS_IMA_T10_MeanAcc	9.95 ± 0.06	9.95 ± 0.05	Mann–Whitney	0.452
TurnPS_IMA_T10_MaxAcc	18.53 ± 1.90	18.41 ± 2.62	Mann–Whitney	0.889
TurnPS_IMA_T10_MeanGyr	56.83 ± 10.38	51.97 ± 12.73	t-test	**0.044**
TurnPS_IMA_T10_MaxGyr	159.83 ± 40.10	146.08 ± 43.25	Mann–Whitney	0.056
TurnPS_IMA_T10_RMSAcc	10.08 ± 0.10	10.07 ± 0.10	Mann–Whitney	0.836
TurnPS_IMA_T10_RMSGyr	67.88 ± 12.61	61.54 ± 14.68	t-test	**0.026**
TurnPS_IMA_T10_SampEnAcc	0.03 ± 0.01	0.03 ± 0.01	t-test	0.302
TurnPS_IMA_T10_SampEnGyr	0.60 ± 0.09	0.61 ± 0.07	Mann–Whitney	0.284
TurnPS_OMA_MELB_Maxjerk	0.02 ± 0.01	0.02 ± 0.01	Mann–Whitney	0.071
TurnPS_OMA_MELB_MaxAngVelJerk	0.36 ± 0.09	0.33 ± 0.09	Mann–Whitney	0.096
TurnPS_OMA_MELB_MeanAcc	10.16 ± 0.16	10.13 ± 0.14	Mann–Whitney	0.275
TurnPS_OMA_MELB_MaxAcc	15.29 ± 1.09	14.86 ± 1.12	t-test	0.057
TurnPS_OMA_MELB_MeanGyr	83.13 ± 22.09	77.80 ± 22.13	t-test	0.234
TurnPS_OMA_MELB_MaxGyr	231.95 ± 60.77	203.97 ± 45.67	t-test	**0.009**
TurnPS_OMA_MELB_RMSAcc	10.26 ± 0.19	10.22 ± 0.16	Mann–Whitney	0.313
TurnPS_OMA_MELB_RMSGyr	94.57 ± 23.71	87.46 ± 23.09	t-test	0.134
TurnPS_OMA_MELB_SampEnAcc	0.00 ± 0.00	0.00 ± 0.00	Mann–Whitney	0.533

TurnPS_OMA_MELB_SampEnGyr	0.80 ± 0.16	0.80 ± 0.11	Mann–Whitney	0.538
TurnPS_OMA_LELB_Maxjerk	0.03 ± 0.01	0.03 ± 0.01	t-test	0.604
TurnPS_OMA_LELB_MaxAngVelJerk	0.35 ± 0.10	0.35 ± 0.10	Mann–Whitney	0.676
TurnPS_OMA_LELB_MeanAcc	10.02 ± 0.10	10.03 ± 0.12	Mann–Whitney	0.981
TurnPS_OMA_LELB_MaxAcc	15.20 ± 1.22	14.85 ± 1.20	Mann–Whitney	0.249
TurnPS_OMA_LELB_MeanGyr	79.19 ± 18.47	74.37 ± 20.18	t-test	0.222
TurnPS_OMA_LELB_MaxGyr	206.93 ± 52.96	195.76 ± 52.62	Mann–Whitney	0.252
TurnPS_OMA_LELB_RMSAcc	10.12 ± 0.13	10.12 ± 0.16	Mann–Whitney	0.965
TurnPS_OMA_LELB_RMSGyr	89.57 ± 20.23	83.96 ± 22.34	t-test	0.199
TurnPS_OMA_LELB_SampEnAcc	0.00 ± 0.00	0.00 ± 0.00	Mann–Whitney	0.746
TurnPS_OMA_LELB_SampEnGyr	0.80 ± 0.14	0.84 ± 0.17	Mann–Whitney	0.139
TurnPS_OMA_MANK_Maxjerk	0.17 ± 0.04	0.18 ± 0.04	Mann–Whitney	0.059
TurnPS_OMA_MANK_MaxAngVelJerk	1.21 ± 0.53	1.47 ± 0.66	Mann–Whitney	**0.008**
TurnPS_OMA_MANK_MeanAcc	12.89 ± 1.03	12.60 ± 1.00	Mann–Whitney	0.336
TurnPS_OMA_MANK_MaxAcc	40.72 ± 9.52	40.92 ± 6.61	Mann–Whitney	0.416
TurnPS_OMA_MANK_MeanGyr	137.93 ± 22.86	131.25 ± 26.28	t-test	0.187
TurnPS_OMA_MANK_MaxGyr	483.68 ± 115.91	485.41 ± 133.77	Mann–Whitney	0.757
TurnPS_OMA_MANK_RMSAcc	14.02 ± 1.45	13.68 ± 1.34	Mann–Whitney	0.444
TurnPS_OMA_MANK_RMSGyr	175.95 ± 27.28	167.87 ± 31.87	t-test	0.186
TurnPS_OMA_MANK_SampEnAcc	0.02 ± 0.01	0.02 ± 0.01	Mann–Whitney	0.552
TurnPS_OMA_MANK_SampEnGyr	0.47 ± 0.14	0.45 ± 0.09	Mann–Whitney	0.711
TurnPS_OMA_LANK_Maxjerk	0.20 ± 0.04	0.21 ± 0.05	t-test	0.689
TurnPS_OMA_LANK_MaxAngVelJerk	1.48 ± 0.86	1.52 ± 0.53	Mann–Whitney	0.086
TurnPS_OMA_LANK_MeanAcc	12.45 ± 0.88	12.02 ± 0.73	Mann–Whitney	**0.036**
TurnPS_OMA_LANK_MaxAcc	42.59 ± 10.14	41.57 ± 9.04	Mann–Whitney	0.916

TurnPS_OMA_LANK_MeanGyr	136.68 ± 24.22	125.87 ± 25.89	t-test	0.036
TurnPS_OMA_LANK_MaxGyr	511.26 ± 185.38	449.39 ± 103.00	Mann–Whitney	0.156
TurnPS_OMA_LANK_RMSAcc	13.45 ± 1.20	12.98 ± 1.01	Mann–Whitney	0.106
TurnPS_OMA_LANK_RMSGyr	174.67 ± 30.19	160.74 ± 30.48	t-test	**0.025**
TurnPS_OMA_LANK_SampEnAcc	0.02 ± 0.01	0.02 ± 0.01	Mann–Whitney	0.570
TurnPS_OMA_LANK_SampEnGyr	0.46 ± 0.12	0.41 ± 0.10	Mann–Whitney	0.030
TurnPS_OMA_PSI_Maxjerk	0.06 ± 0.02	0.06 ± 0.02	t-test	0.740
TurnPS_OMA_PSI_MaxAngVelJerk	0.44 ± 0.21	0.39 ± 0.18	Mann–Whitney	0.343
TurnPS_OMA_PSI_MeanAcc	10.00 ± 0.08	9.99 ± 0.08	t-test	0.406
TurnPS_OMA_PSI_MaxAcc	17.24 ± 1.85	17.12 ± 2.44	Mann–Whitney	0.632
TurnPS_OMA_PSI_MeanGyr	59.34 ± 11.50	53.55 ± 13.59	t-test	**0.027**
TurnPS_OMA_PSI_MaxGyr	154.38 ± 34.46	133.88 ± 29.70	t-test	**0.002**
TurnPS_OMA_PSI_RMSAcc	10.12 ± 0.12	10.10 ± 0.13	Mann–Whitney	0.409
TurnPS_OMA_PSI_RMSGyr	70.23 ± 13.53	62.46 ± 15.54	t-test	**0.010**
TurnPS_OMA_PSI_SampEnAcc	0.02 ± 0.01	0.02 ± 0.01	Mann–Whitney	0.080
TurnPS_OMA_PSI_SampEnGyr	0.58 ± 0.09	0.59 ± 0.09	Mann–Whitney	0.783
TurnPS_OMA_T10_Maxjerk	0.05 ± 0.02	0.05 ± 0.02	Mann–Whitney	0.706
TurnPS_OMA_T10_MaxAngVelJerk	0.68 ± 0.35	0.72 ± 0.34	Mann–Whitney	0.300
TurnPS_OMA_T10_MeanAcc	9.96 ± 0.06	9.96 ± 0.05	Mann–Whitney	0.773
TurnPS_OMA_T10_MaxAcc	18.75 ± 2.33	18.46 ± 2.74	Mann–Whitney	0.726
TurnPS_OMA_T10_MeanGyr	58.84 ± 11.23	53.64 ± 13.91	t-test	**0.048**
TurnPS_OMA_T10_MaxGyr	162.99 ± 42.82	149.39 ± 49.28	Mann–Whitney	0.102
TurnPS_OMA_T10_RMSAcc	10.09 ± 0.11	10.09 ± 0.11	Mann–Whitney	0.938
TurnPS_OMA_T10_RMSGyr	70.12 ± 13.49	63.15 ± 16.12	t-test	0.024
TurnPS_OMA_T10_SampEnAcc	0.03 ± 0.02	0.03 ± 0.02	t-test	0.117

TurnPS_OMA_T10_SampEnGyr	0.58 ± 0.09	0.59 ± 0.08	t-test	0.471
FW_MELB_Maxjerk	0.03 ± 0.01	0.02 ± 0.01	Mann–Whitney	0.230
FW_MELB_MaxAngVelJerk	0.38 ± 0.10	0.35 ± 0.09	Mann–Whitney	0.230
FW_MELB_MeanAcc	10.18 ± 0.13	10.16 ± 0.14	Mann–Whitney	0.641
FW_MELB_MaxAcc	15.57 ± 1.09	15.36 ± 1.48	Mann–Whitney	0.272
FW_MELB_MeanGyr	81.94 ± 24.01	76.59 ± 25.48	t-test	0.288
FW_MELB_MaxGyr	218.61 ± 58.59	191.93 ± 51.75	t-test	**0.017**
FW_MELB_RMSAcc	10.30 ± 0.15	10.28 ± 0.17	t-test	0.528
FW_MELB_RMSGyr	92.09 ± 25.72	85.40 ± 26.82	t-test	0.211
FW_MELB_SampEnAcc	0.01 ± 0.02	0.01 ± 0.01	Mann–Whitney	0.152
FW_MELB_SampEnGyr	0.85 ± 0.14	0.87 ± 0.15	Mann–Whitney	0.287
FW_LELB_Maxjerk	0.03 ± 0.01	0.03 ± 0.01	Mann–Whitney	0.589
FW_LELB_MaxAngVelJerk	0.38 ± 0.11	0.39 ± 0.10	Mann–Whitney	0.182
FW_LELB_MeanAcc	10.10 ± 0.13	10.11 ± 0.16	Mann–Whitney	0.878
FW_LELB_MaxAcc	15.56 ± 1.17	15.25 ± 1.42	Mann–Whitney	0.255
FW_LELB_MeanGyr	79.00 ± 23.05	75.19 ± 25.11	Mann–Whitney	0.512
FW_LELB_MaxGyr	203.39 ± 55.25	202.13 ± 59.22	t-test	0.913
FW_LELB_RMSAcc	10.23 ± 0.16	10.23 ± 0.20	Mann–Whitney	0.970
FW_LELB_RMSGyr	89.11 ± 25.06	85.58 ± 27.58	t-test	0.511
FW_LELB_SampEnAcc	0.01 ± 0.01	0.01 ± 0.01	Mann–Whitney	0.560
FW_LELB_SampEnGyr	0.86 ± 0.18	0.89 ± 0.18	Mann–Whitney	0.260
FW_MANK_Maxjerk	0.16 ± 0.03	0.18 ± 0.04	Mann–Whitney	**0.010**
FW_MANK_MaxAngVelJerk	1.10 ± 0.50	1.25 ± 0.52	Mann–Whitney	**0.028**
FW_MANK_MeanAcc	13.06 ± 1.07	12.75 ± 1.04	Mann–Whitney	0.290
FW_MANK_MaxAcc	39.34 ± 9.62	40.89 ± 8.16	Mann–Whitney	0.117

FW_MANK_MeanGyr	142.07 ± 18.76	133.96 ± 23.77	Mann–Whitney	0.141
FW_MANK_MaxGyr	440.16 ± 115.75	429.78 ± 128.25	Mann–Whitney	0.617
FW_MANK_RMSAcc	14.32 ± 1.53	14.00 ± 1.50	Mann–Whitney	0.622
FW_MANK_RMSGyr	174.38 ± 22.92	165.74 ± 29.22	Mann–Whitney	0.193
FW_MANK_SampEnAcc	0.02 ± 0.01	0.02 ± 0.01	Mann–Whitney	0.841
FW_MANK_SampEnGyr	0.51 ± 0.13	0.50 ± 0.12	t-test	0.776
FW_LANK_Maxjerk	0.15 ± 0.03	0.17 ± 0.04	Mann–Whitney	**0.004**
FW_LANK_MaxAngVelJerk	1.19 ± 0.72	1.34 ± 0.62	Mann–Whitney	**0.008**
FW_LANK_MeanAcc	13.12 ± 0.99	12.70 ± 0.93	t-test	**0.032**
FW_LANK_MaxAcc	38.70 ± 9.40	40.29 ± 9.03	Mann–Whitney	0.293
FW_LANK_MeanGyr	145.56 ± 21.72	136.42 ± 26.17	Mann–Whitney	0.148
FW_LANK_MaxGyr	454.35 ± 180.62	443.40 ± 138.40	Mann–Whitney	0.878
FW_LANK_RMSAcc	14.30 ± 1.40	13.88 ± 1.33	t-test	0.131
FW_LANK_RMSGyr	178.10 ± 27.56	168.06 ± 30.84	Mann–Whitney	0.225
FW_LANK_SampEnAcc	0.02 ± 0.01	0.02 ± 0.01	Mann–Whitney	0.241
FW_LANK_SampEnGyr	0.51 ± 0.16	0.47 ± 0.12	Mann–Whitney	0.293
FW_PSI_Maxjerk	0.06 ± 0.02	0.06 ± 0.02	t-test	0.439
FW_PSI_MaxAngVelJerk	0.48 ± 0.30	0.39 ± 0.17	Mann–Whitney	0.080
FW_PSI_MeanAcc	10.03 ± 0.08	10.01 ± 0.08	t-test	0.195
FW_PSI_MaxAcc	17.66 ± 2.09	17.36 ± 2.43	Mann–Whitney	0.477
FW_PSI_MeanGyr	38.98 ± 10.71	32.73 ± 9.09	t-test	**0.002**
FW_PSI_MaxGyr	137.44 ± 39.42	110.32 ± 33.42	t-test	**<0.001**
FW_PSI_RMSAcc	10.19 ± 0.15	10.16 ± 0.15	Mann–Whitney	0.258
FW_PSI_RMSGyr	48.04 ± 13.09	39.33 ± 11.56	t-test	**0.001**
FW_PSI_SampEnAcc	0.07 ± 0.03	0.08 ± 0.04	Mann–Whitney	0.057

FW_PSI_SampEnGyr	0.76 ± 0.12	0.79 ± 0.15	t-test	0.289
FW_T10_Maxjerk	0.05 ± 0.02	0.05 ± 0.02	Mann–Whitney	0.666
FW_T10_MaxAngVelJerk	0.69 ± 0.40	0.73 ± 0.36	Mann–Whitney	0.303
FW_T10_MeanAcc	9.96 ± 0.07	9.96 ± 0.05	Mann–Whitney	0.637
FW_T10_MaxAcc	18.96 ± 2.38	19.13 ± 3.13	Mann–Whitney	0.742
FW_T10_MeanGyr	35.37 ± 9.75	30.61 ± 8.77	t-test	**0.012**
FW_T10_MaxGyr	148.17 ± 53.51	132.58 ± 51.25	Mann–Whitney	0.193
FW_T10_RMSAcc	10.15 ± 0.15	10.14 ± 0.14	Mann–Whitney	0.905
FW_T10_RMSGyr	46.22 ± 13.83	39.26 ± 12.43	t-test	**0.009**
FW_T10_SampEnAcc	0.08 ± 0.03	0.08 ± 0.03	Mann–Whitney	0.079
FW_T10_SampEnGyr	0.81 ± 0.17	0.82 ± 0.12	Mann–Whitney	0.306
BW_MELB_MaxAcc	15.70 ± 2.51	15.35 ± 2.11	Mann–Whitney	0.731
BW_MELB_MaxAngVelJerk	0.40 ± 0.18	0.37 ± 0.20	Mann–Whitney	0.339
BW_MELB_MaxGyr	187.96 ± 97.03	180.27 ± 85.64	Mann–Whitney	0.884
BW_MELB_Maxjerk	0.03 ± 0.01	0.02 ± 0.01	Mann–Whitney	0.608
BW_MELB_MeanAcc	10.14 ± 0.19	10.12 ± 0.17	Mann–Whitney	0.632
BW_MELB_MeanGyr	67.67 ± 37.79	63.24 ± 31.64	Mann–Whitney	0.747
BW_MELB_RMSAcc	10.26 ± 0.30	10.23 ± 0.25	Mann–Whitney	0.627
BW_MELB_RMSGyr	76.76 ± 42.60	71.67 ± 35.19	Mann–Whitney	0.731
BW_MELB_SampEnAcc	0.01 ± 0.02	0.01 ± 0.01	Mann–Whitney	1.000
BW_MELB_SampEnGyr	0.94 ± 0.22	0.92 ± 0.20	Mann–Whitney	0.686
BW_LELB_Maxjerk	0.03 ± 0.01	0.03 ± 0.01	Mann–Whitney	0.846
BW_LELB_MaxAngVelJerk	0.39 ± 0.21	0.38 ± 0.16	Mann–Whitney	0.796
BW_LELB_MeanAcc	10.06 ± 0.16	10.06 ± 0.17	Mann–Whitney	0.744
BW_LELB_MaxAcc	15.60 ± 2.53	15.35 ± 2.35	Mann–Whitney	0.865

BW_LELB_MeanGyr	62.78 ± 30.83	62.32 ± 29.53	Mann–Whitney	0.984
BW_LELB_MaxGyr	184.12 ± 92.00	180.54 ± 74.23	Mann–Whitney	0.849
BW_LELB_RMSAcc	10.16 ± 0.23	10.17 ± 0.25	Mann–Whitney	0.708
BW_LELB_RMSGyr	71.78 ± 35.50	70.95 ± 32.87	Mann–Whitney	0.997
BW_LELB_SampEnAcc	0.01 ± 0.01	0.01 ± 0.01	Mann–Whitney	0.989
BW_LELB_SampEnGyr	0.93 ± 0.23	0.95 ± 0.28	Mann–Whitney	0.860
BW_MANK_Maxjerk	0.17 ± 0.05	0.16 ± 0.04	Mann–Whitney	0.938
BW_MANK_MaxAngVelJerk	0.92 ± 0.34	1.03 ± 0.44	Mann–Whitney	0.416
BW_MANK_MeanAcc	11.40 ± 0.71	11.19 ± 0.66	Mann–Whitney	0.217
BW_MANK_MaxAcc	35.00 ± 8.64	34.07 ± 9.49	Mann–Whitney	0.409
BW_MANK_MeanGyr	80.27 ± 21.19	73.42 ± 22.44	t-test	0.124
BW_MANK_MaxGyr	284.08 ± 74.22	277.53 ± 74.25	Mann–Whitney	0.525
BW_MANK_RMSAcc	12.13 ± 1.09	11.87 ± 1.14	Mann–Whitney	0.275
BW_MANK_RMSGyr	99.97 ± 26.10	91.75 ± 27.76	t-test	0.135
BW_MANK_SampEnAcc	0.04 ± 0.02	0.04 ± 0.02	t-test	0.921
BW_MANK_SampEnGyr	0.60 ± 0.13	0.60 ± 0.13	Mann–Whitney	0.486
BW_LANK_Maxjerk	0.16 ± 0.05	0.16 ± 0.04	Mann–Whitney	0.543
BW_LANK_MaxAngVelJerk	1.09 ± 0.77	0.91 ± 0.28	Mann–Whitney	0.752
BW_LANK_MeanAcc	11.43 ± 0.68	11.22 ± 0.68	Mann–Whitney	0.111
BW_LANK_MaxAcc	33.90 ± 8.48	32.46 ± 9.15	Mann–Whitney	0.230
BW_LANK_MeanGyr	80.80 ± 22.07	71.56 ± 21.67	t-test	**0.038**
BW_LANK_MaxGyr	318.24 ± 125.76	270.38 ± 64.65	Mann–Whitney	0.175
BW_LANK_RMSAcc	12.06 ± 0.98	11.77 ± 1.04	Mann–Whitney	0.129
BW_LANK_RMSGyr	101.53 ± 26.99	89.62 ± 26.15	t-test	**0.028**
BW_LANK_SampEnAcc	0.03 ± 0.02	0.03 ± 0.01	Mann–Whitney	0.788

BW_LANK_SampEnGyr	0.62 ± 0.15	0.60 ± 0.14	Mann–Whitney	0.646
BW_PSI_Maxjerk	0.04 ± 0.02	0.04 ± 0.02	Mann–Whitney	0.547
BW_PSI_MaxAngVelJerk	0.36 ± 0.37	0.29 ± 0.20	Mann–Whitney	0.244
BW_PSI_MeanAcc	9.98 ± 0.11	9.96 ± 0.08	Mann–Whitney	0.339
BW_PSI_MaxAcc	15.32 ± 2.22	15.04 ± 2.53	Mann–Whitney	0.432
BW_PSI_MeanGyr	22.29 ± 7.28	20.37 ± 6.74	Mann–Whitney	0.191
BW_PSI_MaxGyr	78.52 ± 58.33	64.98 ± 30.03	Mann–Whitney	0.129
BW_PSI_RMSAcc	10.05 ± 0.17	10.02 ± 0.12	Mann–Whitney	0.319
BW_PSI_RMSGyr	25.49 ± 9.33	22.89 ± 7.67	Mann–Whitney	0.115
BW_PSI_SampEnAcc	0.07 ± 0.04	0.06 ± 0.03	Mann–Whitney	0.521
BW_PSI_SampEnGyr	0.91 ± 0.13	0.88 ± 0.13	t-test	0.156
BW_T10_Maxjerk	0.05 ± 0.02	0.04 ± 0.02	Mann–Whitney	0.617
BW_T10_MaxAngVelJerk	0.52 ± 0.39	0.51 ± 0.30	Mann–Whitney	0.473
BW_T10_MeanAcc	9.94 ± 0.06	9.94 ± 0.04	Mann–Whitney	0.323
BW_T10_MaxAcc	16.19 ± 2.20	15.72 ± 2.38	Mann–Whitney	0.217
BW_T10_MeanGyr	23.85 ± 6.67	23.58 ± 8.09	t-test	0.860
BW_T10_MaxGyr	104.19 ± 61.19	103.62 ± 53.92	Mann–Whitney	0.815
BW_T10_RMSAcc	10.01 ± 0.09	10.00 ± 0.09	Mann–Whitney	0.911
BW_T10_RMSGyr	27.54 ± 8.49	26.87 ± 9.61	t-test	0.721
BW_T10_SampEnAcc	0.07 ± 0.04	0.06 ± 0.03	Mann–Whitney	0.202
BW_T10_SampEnGyr	0.92 ± 0.12	0.88 ± 0.11	t-test	0.065

The data are presented as mean ± standard deviation. Statistically significant differences between groups are shown in bold ($p < 0.05$).

TurnFS_IMA, 360° turns at maximum speeds in the direction of the inner step of the more-affected side; TurnFS_OMA, 360° turns at maximum speeds in the direction of the outer step of the more-affected side; TurnPS_IMA, 360° turns at the preferred speed in the direction of the inner step of the more affected side; TurnPS_OMA, 360° turns at preferred speeds in the direction of the outer step of the more-affected side; FW, Forward walking; BW, Backward walking; WS, Walking speed; SLM, Stride length of the more affected side; SLL, Stride length of the less affected side; DSPM, Double-support phase of the more affected side; DSPL, Double-support phase of the less affected side; CTM, More affected–less affected side contralateral temporal coordination; CTL, Less

affected–more affected side contralateral temporal coordination; MELB, More affected side of elbow; LELB, Less affected side of the elbow; MANK, More affected side of the ankle; LANK, Less affected side of the ankle; T10, 10th thoracic spine; PSI, Center of the left and right posterior superior iliac spine; Maxjerk, Maximum jerk; MaxAngVelJerk, Maximum angular velocity jerk; MeanAcc, Mean acceleration; MaxAcc, Maximum acceleration; MeanGyr, Mean gyroscope values; MaxGyr, Maximum gyroscope values; RMSAcc, Root mean square acceleration; RMSGyr, Root mean square gyroscope values; SampEnAcc, Sample entropy of acceleration; SampEnGry; Sample entropy of gyroscope.
