# Supplementary material for: Motor-Derived Digital Biomarkers for Identifying Low-MoCA Status in People with Parkinson’s Disease
Source: Sensors (Basel). 2026 Apr 18;26(8):2503. doi: 10.3390/s26082503 (PMC13119813; doi:10.3390/s26082503)
Supplement: Supplementary file 1 [file sensors-26-02503-s001.zip › Supplementary Table S2.pdf]

Supplementary Table S2. Univariate regression analysis of multi-features associated with MoCA scores

Feature	*β* (SE)	t	*p*-value	*Adjust* R^2
Hoehn and Yahr scale	−0.199(0.090)	−2.217	0.029	0.211
UPDRS total	−0.284(0.090)	−3.175	0.002	0.249
UPDRS Part III	−0.313(0.088)	−3.561	0.001	0.267
SPPB	0.190(0.095)	1.998	0.048	0.204
Mini-BEST	0.239(0.103)	2.319	0.023	0.214
TurnFS_IMA_WS	0.244(0.093)	2.622	0.010	0.226
TurnFS_IMA_SLM	0.219(0.091)	2.422	0.017	0.218
TurnFS_IMA_CTL	−0.203(0.101)	−2.010	0.047	0.204
TurnFS_OMA_WS	0.211(0.095)	2.231	0.028	0.211
TurnFS_OMA_SLL	0.192(0.093)	2.054	0.043	0.206
TurnPS_IMA_WS	0.221(0.094)	2.347	0.021	0.216
TurnPS_IMA_SLM	0.290(0.090)	3.207	0.002	0.250
TurnPS_IMA_SLL	0.200(0.096)	2.082	0.040	0.206
TurnPS_IMA_CTL	−0.197(0.097)	−2.035	0.045	0.205
FW_WS	0.194(0.097)	2.000	0.048	0.204
TurnFS_OMA_MANK_MaxAcc	0.188(0.092)	2.049	0.043	0.205
FW_MELB_MaxGyr	0.192(0.094)	2.049	0.043	0.205
FW_LANK_MaxJerk	−0.232(0.089)	−2.597	0.011	0.225
FW_PSI_RMSGyr	0.205(0.098)	2.093	0.039	0.207

SE, Standard error; MoCA, Montreal Cognitive Assessment; UPDRS, Unified Parkinson's Disease Rating Scale; SPPB, Short Physical Performance Battery; Mini-BEST, Mini-Balance Evaluation Systems Test; TurnFS_IMA, 360° turns at maximum speeds in the direction of the inner step of the more-affected side; TurnFS_OMA, 360° turns at maximum speeds in the direction of the outer step of the more-affected side; TurnPS_IMA, 360° turns at the preferred speed in the direction of the inner step of the more affected side; TurnPS_OMA, 360° turns at preferred speeds in the direction of the outer step of the more-affected side; FW, Forward walking; WS, Walking speed; SLM, Stride length of the more affected side; CTL, Less affected–more affected side contralateral temporal coordination; SLL, Stride length of the less affected side; MANK, More affected side of the ankle; MELB, More affected side of elbow; LANK, Less affected side of ankle; PSI, Center of the left and right posterior superior iliac spine; MaxAcc, Maximum acceleration; MaxGyr, Maximum gyroscope values; MaxJerk, Maximum jerk; RMSGyr, Root mean square gyroscope values.
