# Supplementary material for: Motor-Derived Digital Biomarkers for Identifying Low-MoCA Status in People with Parkinson’s Disease
Source: Sensors (Basel). 2026 Apr 18;26(8):2503. doi: 10.3390/s26082503 (PMC13119813; doi:10.3390/s26082503)
Supplement: Supplementary file 1 [file sensors-26-02503-s001.zip › Supplementary Table S3.pdf]

Supplementary Table S3 Baseline comparison of clinical-only and gait/sensor-only models for evaluating the incremental value of sensor-derived features in classifying low-MoCA status.

**Model**	**Accuracy (mean ± SD)**	**ROC–AUC (mean ± SD)**	**Sensitivity (95% CI)**	**Specificity (95% CI)**	**PPV (95% CI)**	**NPV (95% CI)**
Clinical only						
Logistic regression	0.695 ± 0.137	0.821 ± 0.130	0.500 (0.353–0.655)	0.833 (0.729–0.922)	0.677 (0.500–0.839)	0.704 (0.592–0.808)
SVM (linear kernel)	0.706 ± 0.136	0.810 ± 0.153	0.500 (0.349–0.656)	0.850 (0.754–0.933)	0.700 (0.529–0.862)	0.708 (0.600–0.812)
SVM (RBF kernel)	0.645 ± 0.145	0.703 ± 0.134	0.357 (0.217–0.509)	0.850 (0.758–0.933)	0.625 (0.409–0.815)	0.654 (0.544–0.760)
XGBoost	0.646 ± 0.098	0.761 ± 0.084	0.476 (0.333–0.636)	0.767 (0.657–0.860)	0.588 (0.414–0.742)	0.676 (0.561–0.783)
Gait only						
Logistic regression	0.659 ± 0.162	0.714 ± 0.188	0.500 (0.349–0.634)	0.767 (0.656–0.867)	0.600 (0.433–0.750)	0.687 (0.569–0.797)
SVM (linear kernel)	0.649 ± 0.141	0.689 ± 0.184	0.452 (0.303–0.595)	0.783 (0.672–0.879)	0.594 (0.417–0.758)	0.671 (0.561–0.783)
SVM (RBF kernel)	0.687 ± 0.159	0.724 ± 0.201	0.452 (0.308–0.596)	0.850 (0.750–0.934)	0.679 (0.500–0.839)	0.689 (0.573–0.803)
XGBoost	0.665 ± 0.178	0.738 ± 0.180	0.595 (0.451–0.740)	0.717 (0.600–0.825)	0.595 (0.452–0.738)	0.717 (0.606–0.825)

Models were evaluated using a nested cross-validation framework with stratified group-wise splitting to ensure subject-level independence and prevent data leakage. Performance metrics are reported as mean ± standard deviation across folds. Sensitivity, specificity, positive predictive value (PPV), and negative predictive value (NPV) are presented with 95% confidence intervals estimated using bootstrap resampling. These analyses were conducted to assess the independent and complementary contributions of clinical and sensor-derived features in classifying cognitive status.
